# Nightmare disorder shows reduced slow oscillation-dominant spindle coupling in NREM sleep

**DOI:** 10.1038/s44323-026-00094-0

**Published:** 2026-06-22

**Authors:** Istvan Papp, Zoran Cvetkovic, Nazanin Biabani, Jan Rosenzweig, Camilla Speicher, Alessio Delogu, Alexander D. Nesbitt, Panagis Drakatos, Ines R. Violante, Dijana Vilic, Clive Ballard, David O’Regan, Peter J. Goadsby, Ivana Rosenzweig

**Affiliations:** 1 Sleep and Brain Plasticity Centre, Department of Neuroimaging, Institute of Psychiatry, Psychology and Neuroscience (IoPPN), King’s College, London, UK; 2 Department of Engineering, King’s College London, London, UK; 3 Department of Basic and Clinical Neuroscience, IoPPN, King’s College London, London, UK; 4 Department of Neurology, Guy’s and St Thomas’ NHS Foundation Trust, London, UK; 5 School of Basic and Medical Biosciences, Faculty of Life Science and Medicine, King’s College, London, UK; 6 Sleep Disorders Centre, NHS Guy’s and St Thomas’ Hospital, London, UK; 7 School of Biomedical Engineering and Imaging Sciences, King’s College London, London, UK; 8 Clinical Scientific Computing & Medical Physics and Clinical Engineering, Guy’s and St Thomas’ NHS Foundation Trust, London, UK; 9 Clinical and Biomedical Sciences, Faculty of Health and Life Sciences, University of Exeter, Exeter, UK; 10 NIHR King’s Clinical Research Facility and SLaM Biomedical Research Centre, The Wolfson Sensory, Pain and Regeneration Research Centre, Institute of Psychiatry, Psychology and Neuroscience, King’s College London, King’s College Hospital, Wellcome Foundation Building, London, UK; 11 King Abdullah University of Science and Technology, Thuwal, Saudi Arabia

**Keywords:** Neuroscience, Psychology, Psychology

## Abstract

Nightmare disorder is historically conceptualised as a rapid eye movement (REM) sleep parasomnia, but evidence also points to altered non-rapid eye movement (NREM) sleep physiology. Here, in a retrospective case-control study, we analysed overnight polysomnography from 26 adults with nightmare disorder and 32 controls using a harmonised event-based EEG pipeline. Nightmare disorder showed reduced frontal slow-oscillation–spindle coupling, whereas delta–spindle coupling was preserved, yielding lower coupling dominance than in controls (0.137 ± 0.083 versus 0.238 ± 0.096; *p* = 6.8 × 10^−5^). Stage-adjusted K-complex density across the first 6 h from sleep onset was also lower in nightmare disorder (0.463 ± 0.345 versus 0.723 ± 0.475 events per minute of available N2/N3 sleep; *p* = 0.019), whereas peak-timing differences were not robust. These findings suggest altered NREM sleep microarchitecture in nightmare disorder rather than a phenotype confined to REM sleep, and warrant prospective confirmation in harmonised, clinically phenotyped cohorts.

## Introduction

Nightmares occupy a curious place in the nosology of sleep and psychiatry: they are common phenomena, often dismissed as benign accompaniments of stress or imagination, yet in a significant minority they reach the threshold of nightmare disorder, with recurrent vividly recalled dreams that cause distress, sleep disruption and functional impairment^1^. Clinical nightmare disorder is therefore not interchangeable with frequent distressing dreams reported in population cohorts, even though the two constructs partly overlap.

For many years, nightmare disorder was regarded essentially as a REM-sleep parasomnia, arising from the limbic excesses of REM sleep while leaving the slow rhythms of NREM sleep largely intact^2^. However, polysomnographic work over the past decade has steadily eroded this view^3^. Individuals with frequent nightmares show greater sleep-state instability, altered cyclic alternating pattern (CAP) expression, increased arousals and signs of cortical hyperarousal across NREM as well as REM sleep^4–6^.

Beyond these markers of instability, a small but growing literature has examined spindle activity and stage-N2 microarchitecture in frequent nightmare recallers. Picard-Deland and colleagues reported altered spindle composition, including differences in slow and fast spindle characteristics and links between spindle measures, psychopathology and dream affect^7,8^. Other work has described altered interoceptive and autonomic indices during REM sleep in nightmare disorder^2,9^. Taken together, these findings suggest that nightmare disorder may involve prolonged nocturnal instability spanning both NREM and REM sleep rather than a focal REM sleep-only abnormality.

In parallel, epidemiological work in population cohorts has raised the possibility that frequent distressing dreams may track later neurological risk. In large community samples, more frequent distressing dreams have been associated with later cognitive decline and incident dementia^10,11^. Those studies are important motivation, but they assessed distressing dreams in general-population samples rather than polysomnography-confirmed clinical nightmare disorder. Any bridge between those epidemiological findings and the physiology of diagnosed nightmare disorder, therefore, remains provisional.

If distressing dreams mark an elevated risk of adverse cognitive outcomes, one obvious question is which features of sleep physiology might be relevant. Here, the broader literature on NREM sleep oscillations, memory and ageing becomes pertinent. Slow oscillations ( < 1 Hz), delta waves (1–4 Hz) and thalamocortical spindles (11–16 Hz) support systems consolidation and synaptic homeostasis^12–14^. Sleep spindles are brief, waxing-and-waning sigma-band bursts generated by reciprocal thalamic reticular, thalamocortical and corticothalamic interactions; their frequency, topography and alignment to the depolarising slow-oscillation up-state are thought to provide a temporal scaffold for coordinated NREM sleep communication and memory-related plasticity. In older adults and mild cognitive impairment, these rhythms become shallower and less well coordinated: slow-wave-spindle synchrony is reduced, spindles are less precisely nested in slow-oscillation (SO) up-states, and these changes track overnight forgetting^15–17^.

More recently, slow oscillations and delta waves have been argued to support partially competing operations during NREM sleep^18,19^. Across this framework, spindle events aligned with classical slow oscillations are more closely linked to consolidation, whereas spindle events aligned with faster delta-range activity may index different or even opposing processing demands^16,18,19^. Human nap studies and work in older adults now support a similar competitive picture between SO-spindle and delta-spindle coupling^16^. These considerations make nightmare disorder an interesting model in which to test whether NREM sleep coordination is shifted away from SO-dominant spindle timing.

Against this background, nightmare disorder offers a clinically relevant vantage point. For instance, prior polysomnography (PSG) work indicates enhanced NREM sleep instability, altered spindle expression, and hyperarousal in people with frequent nightmares^4–7^. However, the organisation of slow oscillations, delta waves, spindles and K-complexes has not yet been examined in nightmare disorder within an SO-delta competition framework. Nor has any study, to our knowledge, asked whether the NREM sleep physiology of nightmare disorder resembles a state of reduced SO-dominance relative to controls.

K-complexes are also biologically relevant in this setting because they are prominent N2 events linked to sensory gating, cyclic alternating pattern dynamics and arousal regulation^20,21^. Physiologically, K-complexes are large, transient NREM sleep waveforms that may arise spontaneously or in response to sensory or internal perturbations, and are commonly interpreted as sleep-protective events at the boundary between arousal and sleep preservation. Given prior evidence that nightmare disorder is associated with NREM sleep instability and altered arousal expression^4–6^, examining the nocturnal distribution of K-complexes provides a physiologically motivated way to ask whether sleep-protective NREM sleep microevents are deployed differently across the night.

**Fig. 1:**
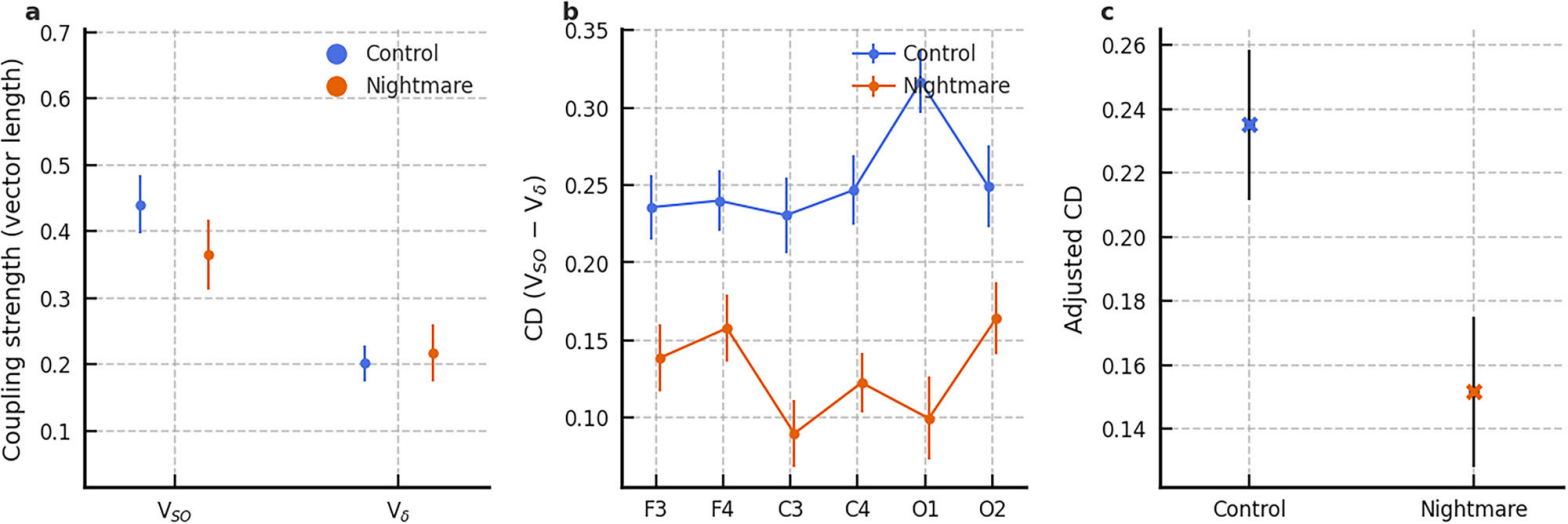
Frontal SO-spindle versus delta-spindle coupling in nightmare disorder. **a** Frontal slow-oscillation-spindle (V_SO) and delta-spindle (V_delta) coupling strength (vector length) averaged across F3/F4. Points show individual subjects (blue, controls; orange, nightmare-disorder patients) with group means ± 95% confidence intervals (black). V_SO is significantly reduced in patients, whereas V_delta is comparable across groups. **b** Channel-wise coupling-dominance index CD = V_SO - V_delta for each scalp site (F3, F4, C3, C4, O1, O2). Lines show group means ± standard error. In controls, CD is strongly positive and largest over frontal-occipital channels; in patients, CD is reduced at all sites (remaining positive on average), indicating a shift away from SO-dominant coupling. **c** Age- and sex-adjusted frontal CD from an ordinary least-squares model (CD ~ Group + Age + Sex). Points show adjusted group means (blue, controls; orange, nightmare-disorder patients) with approximate 95% confidence intervals (black). Coupling dominance remains significantly lower in patients after adjustment, consistent with a stable reduction in SO-dominant spindle coupling in nightmare disorder.

**Fig. 2:**
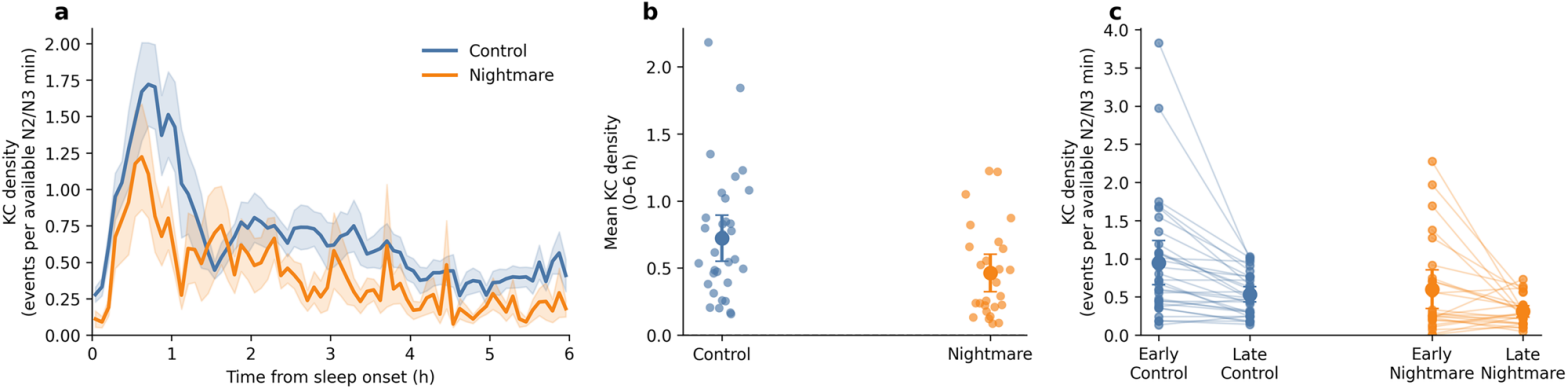
K-complex density in nightmare disorder. **a** Group-mean K-complex density expressed as events per available N2/N3 minute as a function of time from sleep onset, averaged across all six scalp channels for the first 0–6 h. Curves show means (blue, controls; orange, nightmare-disorder patients) with shaded 95% confidence intervals. **b** Subject-level mean K-complex density across 0–6 h from sleep onset, computed as total K-complex count across all six channels divided by total available N2/N3 minutes within the same window. Points show individual subjects (jittered horizontally) with coloured group means ± 95% confidence intervals. **c** Early (0–2 h) and late (2–6 h) stage-adjusted K-complex density for each subject, derived from the six-channel mean density series. Light lines link early and late values within individuals and coloured points show group means ± 95% confidence intervals. Both groups show the expected decline across the night, with lower overall K-complex expression in nightmare disorder.

Here we address these questions by analysing overnight PSG from adults with nightmare disorder and healthy controls using a harmonised event-based pipeline that quantifies slow oscillations, delta waves, spindles, K-complexes and their temporal relationships^16,22^. Given the retrospective design, we treated these analyses as related, theory-informed questions rather than strictly confirmatory tests. *First*, is the balance of SO-linked versus delta-linked spindle coupling reduced in nightmare disorder (Fig. 1)? *Second*, when K-complexes are expressed relative to the available amount of N2/N3 sleep from sleep onset, is their overall expression or timing altered across the first 6 h of the night (Fig. 2)? *Third*, in exploratory supplementary analyses, do age-referenced coupling indices place some nightmare-disorder patients below age-expected SO-dominance?

## Results

We analysed 26 nightmare-disorder patients and 32 controls. Participant demographics and sleep macrostructure are summarised in Table 1. Age did not differ significantly between groups (44.5 ± 17.2 vs 37.7 ± 12.7 years; *p* = 0.086), whereas sex (female, F; male, M) distribution did (16 F/16 M vs 21 F/5 M; *p* = 0.032). Nightmare disorder was additionally associated with lower sleep efficiency, longer REM sleep latency, lower REM sleep proportion and higher arousal index (Table 1). Because patients were drawn from a clinical PSG cohort and controls from an archival research dataset, site and diagnostic group were confounded by design; we therefore emphasise within-recording coupling contrasts and treat absolute spectral measures more cautiously.

**Table 1:** **Participants**.Descriptive statistics (mean ± SD) for demographics and sleep macrostructure variables in controls and nightmare-disorder patients, with p-values from two-sample t-tests (Welch) for continuous variables and chi-square test for sex distribution

Variable	Controls (*n* = 32) Mean ± SD	Patients (*n* = 26) Mean ± SD	*p*-value
Age (years)	44.5 ± 17.2	37.7 ± 12.7	0.086
Total sleep time (min)	421.4 ± 37.5	389.1 ± 75.9	0.055
Sleep efficiency (%)	84.9 ± 7.8	78.3 ± 12.6	0.026
Sleep latency (min)	20.8 ± 12.8	34.3 ± 49.3	0.184
REM latency (min)	77.8 ± 28.5	150.2 ± 95.8	0.001
N1 (% of TST)	8.6 ± 3.9	10.9 ± 5.7	0.093
N2 (%)	46.3 ± 7.1	49.4 ± 12.1	0.255
N3 (%)	21.9 ± 5.4	24.0 ± 10.9	0.362
REM (%)	23.2 ± 4.7	15.7 ± 7.4	<0.001
Apnea–hypopnea index (events/h)	1.8 ± 0.8	3.1 ± 5.3	0.262
Arousal index (events/h)	11.3 ± 3.9	20.6 ± 10.9	<0.001
Sex (F/M)	16 / 16	21 / 5	0.032

Additional definitions, methodological details, and extended results are provided in the Supplementary Information (Supplementary Tables S1–S15; Supplementary Figs. S1–S11).

As a basic EEG overview, we additionally present simple stage-wise Welch power spectra for NREM (N2 + N3) and REM in Supplementary Fig. S10. Because absolute spectral amplitudes are more sensitive to acquisition and site differences than within-recording coupling metrics, we treat these PSD results as descriptive context rather than as primary inferential endpoints.

Coupling strength was quantified as phase–amplitude coupling (PAC) between slow-wave phase and sigma-band amplitude, measured via the mean resultant vector length (MRVL; V). In this framework, coupling strength captures temporal precision: higher V indicates that spindle-amplitude peaks recur at a consistent phase of the slower wave, whereas lower V indicates more dispersed timing; functionally, reduced SO-dominant coupling is best interpreted as less precise recruitment of thalamocortical spindle activity into SO-defined excitable windows. Figure 1a illustrates frontal SO–spindle (V_SO) and delta–spindle (V_δ) coupling strengths averaged across F3/F4. Controls showed stronger SO–spindle coupling than patients (V_SO: 0.443 ± 0.127 vs 0.356 ± 0.136; *d*= −0.66, *p* = 0.016), whereas delta–spindle coupling was similar (V_δ: 0.204 ± 0.079 vs 0.219 ± 0.112; *d* = 0.16, p = 0.56).

The coupling dominance index CD = V_SO − V_δ was therefore substantially reduced in patients (Fig. 1b). Frontal CD was 0.238 ± 0.096 in controls and 0.137 ± 0.083 in patients (*d* ≈ −1.12, *p* ≈ 6.8×10⁻⁵). In LMMs adjusting for Age, Sex and Channel, Group (Nightmare vs Control) remained a strong predictor of CD (*β* = −0.12, *p* < 10⁻⁷) and V_SO (*β* = −0.11, *p* = 10⁻⁴), but not V_δ (*β* = +0.01, p = 0.7). Thus, nightmare disorder is characterised by a selective weakening of SO–spindle coupling and consequent loss of SO-dominant spindle engagement, rather than by increased delta–spindle coupling. Representative time–frequency and phase-coupling profiles are shown in Supplementary Fig. S2.

Using a bias-resistant estimator (pairwise phase consistency; PPC), we observed the same pattern as for vector length: frontal PPC_SO was lower in nightmare disorder (0.206 ± 0.116 in controls vs 0.134 ± 0.102 in patients; Welch *p* = 0.0143), whereas PPC_ δ was comparable (0.046 ± 0.035 vs 0.058 ± 0.054; Welch *p* = 0.328). Accordingly, PPC-based coupling dominance (PPC_CD = PPC_SO - PPC_ δ) remained markedly reduced in patients (0.160 ± 0.095 vs 0.075 ± 0.068; Welch *p* = 2.23e-04; Supplementary Table S12). To further assess whether demographic imbalance could explain these effects, we performed an age- and sex-matched control resampling analysis. Across 10,000 matched resamples, both CD and PPC_CD remained lower in nightmare-disorder patients (direction consistent in 100% of resamples), with median effect sizes in the large (CD) and moderate-to-large (PPC_CD) range (Supplementary Table S13; Supplementary Fig. S8). In this multivariable model, Group remained associated with lower frontal CD (patient-control coefficient −0.088; *p* = 0.020), suggesting that reduced SO-dominant coupling is not fully accounted for by differences in macrostructure or arousal burden. However, because arousal index and sleep efficiency may lie on the causal pathway from nightmare disorder to altered coupling, this adjustment should be interpreted as a robustness check rather than as a claim of complete mechanistic independence (Supplementary Table S15).

In frontal channels (F3/F4), the SO/delta nesting ratio NestRatio = N_SO/N_ δ was below 1 in both groups, indicating that delta-nested spindles were more frequent than SO-nested spindles overall. Using subject-level frontal NestRatio values (averaged across F3 and F4 for each subject), mean ± SD NestRatio was 0.274 ± 0.105 in controls (*n* = 32) and 0.247 ± 0.104 in nightmare disorder (*n* = 26). The between-group difference was modest and non-significant (Welch *p* = 0.342, Cohen’s *d* = −0.25), consistent with the view that the primary abnormality in nightmare disorder lies in the balance between SO‑ and delta‑related coupling strengths (captured by the coupling‑dominance index, CD) rather than in a wholesale loss of SO‑nested spindles. Delta nesting is common in adults; the key group difference here is coupling dominance (CD), not nesting counts (Supplementary Table S2). Summary statistics are provided in Supplementary Table S4.

Although total spindle burden was higher in nightmare disorder (Supplementary Table S7), spindles were substantially less likely to occur within ±0.5 s of any SO or delta peak in frontal channels. The fraction of frontal spindles nested in any slow-wave event (Prop_nested_any) was 0.394 ± 0.037 in controls versus 0.290 ± 0.059 in nightmare disorder (Welch *p* = 1.66e-09). Conversely, the un-nested fraction (Prop_unnested) was higher in patients (0.710 ± 0.059 vs 0.606 ± 0.037; Welch *p* = 1.66e-09). Consistent with this, the count of un-nested frontal spindles (F3 + F4) was higher in patients (2378 ± 601 vs 1689 ± 344; Welch *p* = 7.19e-06; Supplementary Table S12). Together, these results suggest that increased spindle burden in nightmare disorder is disproportionately expressed as un-nested (mis-timed) spindle events rather than as increased SO- or delta nesting. Of note, no systematic hemispheric lateralisation differences were observed (Supplementary Fig. S3).

Within subjects, background delta power had a robust effect on the SO/delta nesting ratio in both groups. In controls, frontal NestRatio increased from 0.19 ± 0.11 in Low δ epochs to 0.32 ± 0.12 in High δ epochs (*n* = 32, paired *p* = 1.1e-07). A similar pattern was observed in nightmare-disorder patients, where frontal NestRatio rose from 0.13 ± 0.06 in Low δ epochs to 0.30 ± 0.13 in High δ epochs (*n* = 26, paired *p* = 7.9e-08, Supplementary Table S3). Thus, high-delta states were, if anything, associated with relatively more SO-nested spindles in both groups. By contrast, frontal V_SO showed only small, non-significant low–high delta differences in controls (0.45 ± 0.14 vs 0.45 ± 0.13, *p* = 0.74) and in nightmares (0.44 ± 0.15 vs 0.39 ± 0.14, *p* = 0.22). Taken together with Supplementary Fig. S4, these results indicate that high-delta NREM sleep epochs do not selectively abolish SO-nested spindles or SO–spindle coupling in nightmare disorder; rather, the main group difference is a generally reduced SO-dominant regime across contexts.

K-complex analyses were expressed as stage-adjusted density from sleep onset rather than raw counts per clock bin. For each 5-min bin and channel, K-complex density was defined as KC count divided by the available N2/N3 minutes in that bin; the six-channel mean density trajectory is shown in Fig. 2a. Both groups showed the expected higher early-night expression followed by decline across the first 6 h from sleep onset, but nightmare-disorder patients exhibited lower overall K-complex expression throughout much of this window.

At the subject level, mean K-complex density over 0–6 h was lower in nightmare disorder than in controls (0.463 ± 0.345 vs 0.723 ± 0.475 events per available N2/N3 min; *d* = −0.62, *p* = 0.019; Fig. 2b; Supplementary Table S4). The difference was most evident in the later part of the analysed window (Fig. 2c). In OLS models adjusting for Age, Sex and N2N3%, Group remained associated with lower mean K-complex density (*β* = −0.426, SE = 0.117, *p* = 6.3 × 10^−4; Supplementary Table S15; Supplementary Fig. S1). By contrast, KC_tpeak was later numerically but not significantly in nightmare disorder (1.97 ± 1.40 vs 1.63 ± 1.11 h; *p* = 0.326), and KC_time_slope is reported only as a secondary sensitivity metric in the Supplement (Supplementary Figs. S6, S7, S11).

Prespecified sensitivity analyses had negligible impact on the main group differences. Following exclusion, frontal coupling dominance CD changed only slightly (controls 0.238 ± 0.096 vs nightmares 0.137 ± 0.083 in the full sample; controls 0.238 ± 0.096 vs nightmares 0.137 ± 0.085 after exclusion; Supplementary Table S8 and Supplementary Fig. S5). Frontal V_SO remained lower in nightmares both before and after exclusion (controls 0.443 ± 0.127 vs nightmares 0.356 ± 0.136; controls 0.443 ± 0.127 vs nightmares 0.351 ± 0.135 after exclusion). Mean stage-adjusted K-complex density was similarly stable (controls 0.723 ± 0.475 vs nightmares 0.463 ± 0.345 in the full sample; controls 0.723 ± 0.475 vs nightmares 0.475 ± 0.347 after exclusion), while KC_tpeak changed minimally (nightmares 1.97 ± 1.40 h in the full sample vs 1.92 ± 1.41 h after exclusion; Supplementary Table S8 and Supplementary Fig. S7). In addition, the primary coupling results were robust to spindle burden and extreme spindle counts (Supplementary Table S11), and event-count matched resampling (10,000 iterations) produced effect sizes consistent in direction with the full sample (Supplementary Table S14; Supplementary Fig. S9).

## Discussion

Nightmare disorder is clinically defined by distressing dreams, but the present findings suggest that its physiology extends into the architecture of NREM sleep. Rather than indicating a global loss of NREM oscillatory activity, the data point to a selective weakening of slow-oscillation-dominant spindle nesting, alongside preserved delta-linked coupling and lower K-complex expression per available N2/N3 sleep. Foremost, frontal SO-spindle coupling was lower in nightmare disorder, delta-spindle coupling was broadly preserved, and the coupling-dominance index CD = V_SO - V_delta was substantially reduced, with robust support from mixed-effects models, PPC-based analyses and matched-resampling sensitivity tests.

A second result is that spindle timing appears less tightly coordinated with slow-wave events in nightmare disorder. Although total spindle burden was higher in patients, a smaller fraction of frontal spindles occurred within +/−0.5 s of either SO or delta peaks, and the excess spindle burden was disproportionately expressed as un-nested events. Taken together with the coupling findings, this points to altered NREM sleep coordination rather than a simple deficit of spindle generation.

Overall, K-complex density expressed per minute of available N2/N3 sleep from sleep onset was significantly lower in nightmare disorder across the first 6 h of the night. Both groups retained the expected early-night predominance and subsequent decline, but nightmare-disorder patients showed lower mean density, particularly later in the analysed time window.

Earlier PSG microstructure work in frequent nightmare sufferers showed a shift in cyclic alternating pattern (CAP) away from A1, the slower, more sleep-stabilising subtype, and toward the more arousal-like A2/A3 subtypes^5^. K-complexes fit naturally within this framework because, although they are not identical to CAP subtypes^23^, they are among the classic synchronized NREM sleep responses involved in sensory gating and preservation of sleep continuity when the sleeping brain is challenged^24–26^. Seen in that light, the lower stage-adjusted K-complex density observed here is directionally consistent with weaker sleep-protective buffering during NREM sleep, rather than with a uniform increase in all forms of nocturnal activation. In a broader, indirect sense, this places nightmare disorder closer to conditions marked by fewer slow-wave-linked, sleep-stabilising NREM sleep events across the night (e.g. isolated REM behaviour disorder or narcolepsy) than to NREM sleep parasomnia, where early-night phasic activity appears amplified^27,28^. These findings extend prior PSG studies that have described NREM sleep instability, altered CAP expression, increased arousals and spindle abnormalities in frequent nightmares^4–7^, while complementing REM sleep-focused work on autonomic, interoceptive and theta-related alterations^2,9,29^. Our data therefore support a broader view in which nightmare disorder is not only a REM sleep phenomenon, but also involves altered NREM sleep event coordination.

The SO-delta competition framework provides a useful mechanistic context, but it should be treated here as context rather than proof of altered memory processing. Reduced SO-dominant coupling is consistent with work linking degraded SO-spindle synchrony to ageing and impaired memory consolidation^15–17^, and it is tempting to ask whether nightmare disorder occupies part of the same physiological axis. However, the present cohort did not undergo cognitive testing, and no longitudinal outcomes were available; any link to consolidation, forgetting, or dementia risk therefore remains hypothesis-generating rather than demonstrated.

It is equally important not to over-pathologise delta activity or K-complexes. Delta-linked processes and K-complex generation can be adaptive components of normal sleep physiology^16,18–21^. What the present data suggest is not that these phenomena are intrinsically abnormal, but that in nightmare disorder, they occur against a background of weakened SO-dominant coordination and greater NREM sleep instability.

**Fig. 3:**
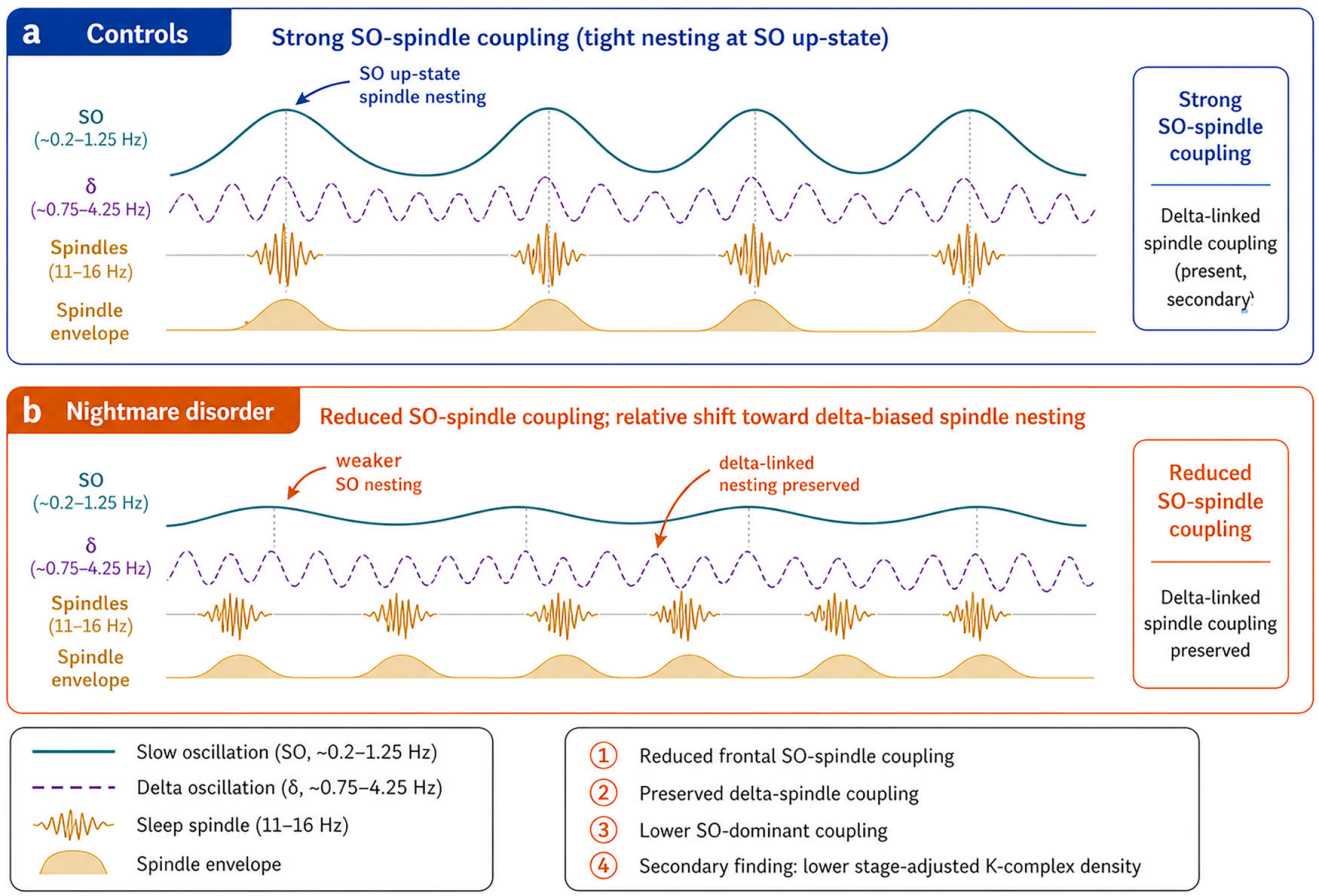
Proposed shift in NREM sleep spindle nesting in nightmare disorder. Conceptual schematic summarising the principal group-level findings. Traces are shown for illustration only and are not drawn to scale. **a** Controls. In controls, sleep spindles (11–16 Hz) are depicted as preferentially nested around the up-state of slow oscillations (SO; ~0.2–1.25 Hz), consistent with stronger frontal SO–spindle coupling and greater SO-dominant coupling. Delta oscillations (δ; ~0.75–4.25 Hz) and delta-linked spindle coupling are also present, but are shown as secondary to SO-linked nesting. **b** Nightmare disorder. In nightmare disorder, frontal SO–spindle coupling is reduced, such that spindle events are depicted as less tightly aligned to the SO up-state and relatively more associated with delta-linked timing, consistent with preserved delta–spindle coupling and reduced overall SO-dominant coupling. The lower panel, therefore, illustrates a relative shift from an SO-dominant to a more delta-biased spindle-nesting regime in nightmare disorder. The spindle envelope is shown beneath each schematic spindle burst to indicate the temporal window of spindle expression. The summary box also notes the secondary finding of lower stage-adjusted K-complex density in nightmare disorder across the first 6 h from sleep onset.

Clinically, these observations suggest that nightmare disorder may involve a measurable NREM sleep coordination phenotype in addition to disturbed dreaming. That proposition remains relevant whether nightmares are idiopathic or occur alongside broader psychiatric or sleep-system comorbidity, although the present sample was not large enough to dissect those subgroups. A simplified schematic summary of the working model and its limits is provided in Fig. 3.

Several notable limitations merit acknowledgement. First, this was a retrospective case-control study using controls from a different acquisition site than the patients; site and diagnostic group were therefore confounded, and residual acquisition- or scoring-related differences cannot be fully excluded despite harmonised offline preprocessing. Secondly, sex distribution differed between groups, although age did not differ significantly; we therefore retained age and sex adjustment and matched-resampling sensitivity analyses, but residual demographic confounding remains possible. Thirdly, nightmare-disorder patients were clinically heterogeneous, and symptom-severity measures were not harmonised for correlation analyses. Finally, we lacked cognitive and longitudinal outcome data, so we cannot determine whether the observed oscillatory phenotype predicts treatment response, daytime function or later cognitive decline.

Notwithstanding these caveats, the present work demonstrates that nightmare disorder is associated with a quantifiable re-organisation of NREM sleep microarchitecture. Reduced SO-dominant spindle coupling was the clearest and most reproducible signal, and lower stage-adjusted K-complex density provided convergent evidence that NREM sleep event expression is altered beyond a purely REM sleep-restricted account. Future prospective studies should test whether these markers track symptom burden, respond to treatment, and relate to next-day cognitive or affective function.

## Methods

We analysed overnight polysomnography (PSG) recordings from 26 adults with nightmare disorder and 32 healthy controls. Nightmare-disorder patients were recruited from the Sleep Disorders Centre at Guy’s and St Thomas’ Hospital (GSTT), London, and met International Classification of Sleep Disorders ICSD-3 criteria^30^ for nightmare disorder following specialist assessment. Controls were drawn from the Montreal Archive of Sleep Studies (MASS)^31^ and were free of major sleep, neurological or psychiatric disorders. All clinical recordings were obtained as part of routine care; retrospective analysis was approved by the local research ethics committee and conducted in accordance with the Declaration of Helsinki^32^. Controls were taken from the publicly available MASS dataset under its original ethical approvals^31^.

Research involving human participants, material and data was performed in accordance with the Declaration of Helsinki. This secondary analysis of de-identified clinical polysomnography records was conducted under the Guy’s and St Thomas’ NHS Foundation Trust Electronic Records Research Interface (GERRI) governance framework. The parent GERRI research database has NHS Research Ethics Committee approval from East Midlands - Leicester Central Research Ethics Committee (IRAS ID 257283; REC reference 20/EM/0112; favourable opinion 11 May 2020). The present project, “Digital Biomarkers of Parasomnias”, was approved by the GERRI Oversight Committee on 10 June 2024. Individual consent for this secondary analysis of de-identified routine clinical records was waived/not required under the approved GERRI governance framework, with records used subject to institutional governance restrictions and applicable opt-out arrangements. For MASS controls, source recordings were sanctioned by the Comité d’éthique de la Recherche du Centre de Recherche de l’Hôpital du Sacré-Coeur de Montréal (Project Ref 2013-935; BQ-935), participants gave written informed consent at original acquisition, and the present analysis followed MASS access and data-use conditions.

Clinical recordings used standard 10-20 EEG (including F3, F4, C3, C4, O1, O2) referenced to contralateral mastoids (M1, M2), with bilateral EOG, submental and tibialis EMG, and ECG. Data were digitised at 512 Hz. MASS recordings used a comparable montage at 256 Hz31. All nights were visually scored in 30-s epochs according to AASM criteria by experienced technologists^33,34^. To harmonise datasets, raw EDF signals were imported into a unified pipeline (MNE-Python and associated libraries^35^). Clinical data were resampled to 256 Hz. EEG channels were re-referenced to averaged mastoids and band-pass filtered (0.2-35 Hz, zero-phase FIR) with notch filters at the local mains frequency (50 Hz at GSTT, 60 Hz in MASS). Automated artefact detection (flatlines, saturations, extreme amplitude) was applied, as previously described^36^, followed by visual QC. Analyses were restricted to NREM sleep stages N2 and N3 and to six homologous EEG channels (F3/F4, C3/C4, O1/O2) present in both datasets. Acquisition and harmonisation details, including original acquisition references and hardware filters, are summarised in Supplementary Table S6.

Sleep spindles were detected per EEG channel using an established RMS‑envelope approach, in line with published NREM event‑detection pipelines^15,16^. EEG was band‑pass filtered at 11–16 Hz (zero‑phase FIR), and the root‑mean‑square (RMS) envelope was computed using 200‑ms windows. Candidate spindle events were defined as contiguous segments of 0.5–3.0 s in which the RMS envelope exceeded a channel‑specific threshold set to the upper quartile (75th percentile) of the within‑night RMS distribution; events were retained only if fully contained within N2/N3 and free of artefact. For each retained spindle, peak‑to‑peak amplitude and dominant frequency (Welch power spectrum) were computed, and events were classified as slow ( < 12.5 Hz) or fast ( ≥ 12.5 Hz) using a fixed cut‑off approach^15,16^. In complementary analyses, individual slow‑ and fast‑sigma peak frequencies were estimated using a multichannel, topography‑informed generalised eigendecomposition procedure^22^, implemented in open‑source sleep‑analysis toolchains^36^. Slow oscillations (SOs) were detected from signals band‑pass filtered at 0.2–1.25 Hz using downward zero‑crossings to define candidate boundaries; events of 1.0–2.0 s were retained, and the top quartile by trough‑to‑peak amplitude was selected^15,16^. Delta waves were detected analogously using a 0.75–4.25 Hz filter and a 0.25–1.0 s duration window, retaining the top quartile by peak‑to‑peak amplitude^16^. The SO (0.2–1.25 Hz) and delta (0.75–4.25 Hz) phase bands partially overlap by design, but the duration criteria sharply separate the detected events (SOs: 1–2 s; delta: 0.25–1 s), so only a narrow set of ~1 Hz waveforms can satisfy both definitions^16^. Consistent with prior work using the same frequency-based approach, any residual overlap is small and physiologically expected (SOs can occasionally group delta activity)^16^. Crucially, spindles are never double-counted: if both an SO and a delta candidate are present around a spindle, it is assigned once to the event whose up-state peak is temporally closest^16^. Moreover, K-complexes were operationalised in accordance with established physiological definitions^21,37^, following a published method^38^.

To quantify the nocturnal time course of K-complex expression, each recording was partitioned into contiguous 5-min clock-time bins referenced to sleep onset. Within each channel and bin, only K-complexes occurring during N2/N3 epochs were counted. For each bin we also recorded the available amount of N2/N3 sleep (minutes; range 0–5). Channel-wise KC density was defined as KC_count / available N2/N3 minutes (events per available N2/N3 min); bins with zero available N2/N3 time were treated as missing. For visualisation, six-channel mean KC_density(t) was obtained by averaging channel-wise densities across F3/F4/C3/C4/O1/O2 over finite bins. Our primary summary measure was mean K-complex density over 0–6 h from sleep onset, computed as the total KC count across all six channels divided by the total available N2/N3 minutes across the same window. Secondary timing metrics were KC_tpeak, defined as the midpoint time of the maximal six-channel mean density bin within 0–6 h, and KC_time_slope, defined as the OLS slope of the six-channel mean density series versus time (hours) across finite bins.

Spindle nesting was performed following previously described approach^16^. To minimise detection of inauthentic cross-frequency coupling, which can be caused by differences in oscillatory power, a normalisation of individual events was conducted to minimise amplitude differences prior to all subsequent analyses^39^, as previously described^16^. Accordingly, spindles thus contributed to either SO-nested (N_SO) or delta-nested (N_δ) counts^16^. Spindles for which no SO or delta event was detected within the ±0.5 s search window were classified as un-nested. In addition to NestRatio (N_SO/N_ δ), we quantified the fraction of spindles nested within any slow-wave event as: Prop_nested_any = (N_SO + N_delta) / N_TotalSpindles, and the complementary un-nested fraction as: Prop_unnested = 1 - Prop_nested_any. Proportions were also computed separately for F3 and F4 channels using that channel’s spindle count as the denominator, then averaged within subject; N_unnested is additionally reported as the pooled F3 + F4 count for scale. In this case, Prop_nested_any = (N_SO + N_δ) / N_TotalSpindles_FrontCh.

These proportions were computed per channel and then averaged across frontal channels (F3/F4) at the subject level for group comparisons (see Supplementary Table S12). All spindle nesting counts (N_SO, N_δ, N_unnested and N_total) were computed at the channel level. For frontal summaries, proportions were computed for each channel (F3 and F4) and then averaged within subject; pooled frontal counts (F3 + F4) are additionally reported for interpretability.

We also defined a coupling dominance index (CD):

$${CD}={V}_{{\rm{SO}}}-{V}_{\delta },$$

where V_SO and V_δ are PAC vector lengths (below).

Time–frequency representations (TFRs) were computed on ±2.5 s segments around SO and delta troughs, using an established approach^15,16^. Power from 5 to 20 Hz was estimated using Hann-tapered convolution with 5 cycles per frequency (time step 10 ms; frequencies sampled in 0.25 Hz steps). Power was baseline-normalised as z-scores relative to a pre-event window ( − 2.5 to −1.25 s). For visualisation (and any inferential testing on TFRs), analyses were restricted to the central ±1.25 s around the trough to minimise edge effects. For each frequency $f$, $Z(f,t)=\frac{P(f,t)-{\mu }_{f}^{{base}}}{{\sigma }_{f}^{{base}}}$, where ${\mu }_{f}^{{base}},{\sigma }_{f}^{{base}}$ are computed over −2.5 to −1.25 s.

Similarly, for phase–amplitude coupling (PAC) we used an established approach^15,16,40^; we extracted ±2.5 s segments and applied separate band-pass filters for phase (SOs: 0.2–1.25 Hz; delta: 0.75–4.25 Hz) and spindle amplitude (11–16 Hz). Instantaneous phase and amplitude were derived via the Hilbert transform, and analysis was restricted to the inner ±2.0 s. For each event, we identified the time of maximal spindle amplitude and its corresponding slow-wave phase. Across events, we computed the circular mean phase and the mean resultant vector length (V) of the slow-wave phase sampled at the sigma-amplitude peak, yielding coupling strengths V_SO and V_δ.

Because mean resultant vector length can show finite-sample bias when the number of events differs across subjects or conditions, we additionally computed pairwise phase consistency (PPC), a bias-resistant phase-locking metric^41^. PPC was derived from the resultant vector length (V) and the number of events (n) as:

$${\rm{PPC}}=\frac{n{V}^{2}-1}{n-1}$$

We computed PPC separately for SO-locked and delta-locked phase distributions (PPC_SO and PPC_ δ), and a PPC-based dominance index PPC_CD = PPC_SO - PPC_ δ (Supplementary Table S12). We additionally derived a slow-oscillation/delta nesting index based on the ratio of SO-nested to delta-nested spindles (NestRatio = N_SO / N_δ) for each subject, channel and delta context. Values yielding infinite or undefined ratios (e.g. N_δ = 0) were set to missing for analysis. Frontal NestRatio values were averaged across F3/F4 channels to obtain a single index per subject and context.

To quantify the influence of background delta power on SO–spindle coupling and nesting, we used the LowDelta and HighDelta strata, as previously argued and described^16,42^. For each subject and frontal channel, V_SO and NestRatio were averaged within LowDelta and HighDelta epochs. Background delta power was estimated for each 30-s N2/N3 epoch as the mean Hilbert-derived amplitude (envelope) of the delta-band filtered signal. Within each subject, epochs were stratified by a median split into LowDelta (below-median delta power) and HighDelta (above-median delta power), and coupling/nesting metrics were recomputed separately within each stratum^16^. We then formed subject-level frontal averages across F3/F4 and performed within-group paired comparisons (HighDelta vs LowDelta) and between-group comparisons at the subject level. Formal statistics for the low–high delta contrasts are reported in the Supplement.

Coupling analyses quantified phase–amplitude coupling using a fixed sigma-band amplitude signal (11–16 Hz) and the phase of SO (0.2–1.25 Hz) or delta (0.75–4.25 Hz) events, yielding resultant vector lengths (V_SO and V_δ) and coupling dominance (CD = V_SO − V_δ). Individual/adaptive peak-frequency estimation was used to characterise spindle subtype parameters (Supplementary Table S10), but coupling was not recomputed using individualized sigma amplitude bands in the present study. However, because individualized slow and fast spindle peaks lie within the sigma range used for amplitude extraction, the primary coupling metrics are expected to be robust to reasonable inter-individual shifts in peak frequency; nevertheless, future analyses should explicitly repeat coupling using individualized sigma bands and test sensitivity to spindle amplitude and duration^43^.

To place individual coupling profiles in an age-referenced context, we fitted linear regressions of frontal CD and V_SO on age in the control group only. For each metric Y (CD or V_SO):

$${Y}_{i}={\beta }_{0}+{\beta }_{1}{\mathrm{Age}}_{i}+{\varepsilon }_{i}.$$

Predicted values were computed for all subjects and residuals were converted to z-scores using the residual SD in controls, yielding CD_{age,z} and V_{SO,age,z}, which index deviation from age-expected SO-dominance.

To provide a simple background EEG overview, we additionally computed stage-wise Welch power spectral density (PSD) estimates after the same harmonised preprocessing. PSD was calculated separately for NREM (N2 + N3 combined) and REM sleep using 4-s Hann windows with 50% overlap, constant detrending and density scaling, and summarised over 0.5-30 Hz. Group curves were averaged across the six homologous EEG channels. Because absolute spectra are more vulnerable to site and acquisition differences than our primary within-recording coupling metrics, PSD results are presented as descriptive supplementary analyses rather than as primary inferential endpoints.

Descriptive statistics are reported as mean ± SD unless otherwise noted. Group differences in frontal coupling metrics (CD, V_SO, V_delta), mean KC density, KC_tpeak and KC_time_slope were first summarised with Cohen’s d and Welch t-tests. To account for repeated channels per subject, we fitted linear mixed-effects models (LMMs) using CD, V_SO or V_delta as the dependent variable, with Group (Control vs Nightmare), Channel, Age and Sex as fixed effects and a random intercept. Site could not be included as a fixed effect because Group and Site were perfectly confounded. Group differences in subject-level mean KC density and secondary KC timing metrics were further tested with OLS models adjusting for Age and Sex, with additional sensitivity models including N2N3% (Supplementary Table S15). Because the cohorts were not prospectively matched and sex distribution differed, we additionally performed an age- and sex-matched control resampling analysis (10,000 iterations) for frontal CD and PPC_CD, matching sex exactly and weighting selection by age proximity (Gaussian kernel; sigma = 10 years; Supplementary Table S13; Supplementary Fig. S8). We also performed an event-count matched resampling analysis for frontal V_SO, CD and PPC_CD to assess potential finite-sample bias in coupling metrics (Supplementary Table S14; Supplementary Fig. S9). Unless specified as exploratory, tests were two-sided and interpreted at alpha = 0.05 without formal multiplicity correction beyond the limited primary questions of reduced SO-dominant coupling and altered K-complex expression.

To assess whether the principal findings were driven by individuals with marked comorbid restless legs/periodic limb movements or heavy sedative/psychotropic medication use, we repeated the primary frontal coupling analyses and predefined K-complex summary analyses after excluding nightmare-disorder participants with substantial sleep-related comorbidity or heavy sedative/psychotropic medication use, defined as a periodic limb movement index >30 events/h and/or regular nightly use of high-dose benzodiazepines or combined benzodiazepine-antidepressant treatment. Only one patient met this criterion. The documented exclusion analyses are reported in Supplementary Table S8 and Supplementary Figs. S5 and S7.

A Zenodo record containing the custom Python analysis code will be made publicly available upon publication, subject to institutional governance restrictions. The analysis used a Python/MNE-Python-based pipeline; software-version information is recorded in the accompanying environment files and will be deposited with the code in the Zenodo record. The event-detection, filtering, coupling and statistical variables and parameters used to generate and analyse the current datasets are specified in the Methods and Supplementary Tables S1 and S6.

## Supplementary information

Supplementary Information

## Data Availability

The datasets generated and/or analysed during the current study are not publicly available because the underlying clinical records and derived individual-level data are subject to governance restrictions under the GSTT Electronic Record Research Interface (GERRI) approval and cannot be transferred outside approved institutional firewalls. De-identified derived summary data sufficient to reproduce the main figures and tables are available from the corresponding author on reasonable request, subject to institutional approvals and a data-sharing agreement where required.

## References

[1] Gieselmann, A. et al. Aetiology and treatment of nightmare disorder: State of the art and future perspectives. *J. Sleep. Res.* **28**, e12820 (2019). 30697860 10.1111/jsr.12820 PMC6850667

[2] Perogamvros, L. et al. Increased heartbeat-evoked potential during REM sleep in nightmare disorder. *NeuroImage: Clin.* **22**, 101701 (2019). 30739843 10.1016/j.nicl.2019.101701 PMC6370851

[3] van der Wijk, G., Blaskovich, B., Farahzadi, Y. & Simor, P. Unaltered EEG spectral power and functional connectivity in REM microstates in frequent nightmare recallers: are nightmares really a REM parasomnia? *Sleep. Med.* **75**, 192–200 (2020). 32858360 10.1016/j.sleep.2020.07.014

[4] Blaskovich, B., Reichardt, R., Gombos, F., Spoormaker, V. I. & Simor, P. Cortical hyperarousal in NREM sleep normalizes from pre- to post- REM periods in individuals with frequent nightmares. *Sleep* **43**, zsz201 (2020). 31556954 10.1093/sleep/zsz201

[5] Simor, P., Bódizs, R., Horváth, K. & Ferri, R. Disturbed dreaming and the instability of sleep: altered nonrapid eye movement sleep microstructure in individuals with frequent nightmares as revealed by the cyclic alternating pattern. *Sleep* **36**, 413–419 (2013). 23449753 10.5665/sleep.2462 PMC3571751

[6] Simor, P., Horváth, K., Ujma, P. P., Gombos, F. & Bódizs, R. Fluctuations between sleep and wakefulness: wake-like features indicated by increased EEG alpha power during different sleep stages in nightmare disorder. *Biol. Psychol.* **94**, 592–600 (2013). 23831546 10.1016/j.biopsycho.2013.05.022

[7] Picard-Deland, C., Carr, M., Paquette, T., Saint-Onge, K. & Nielsen, T. Sleep spindle and psychopathology characteristics of frequent nightmare recallers. *Sleep. Med.* **50**, 113–131 (2018). 30031989 10.1016/j.sleep.2017.10.003

[8] Picard-Deland, C., Carr, M., Paquette, T. & Nielsen, T. Sleep spindles are altered in early- but not late-onset nightmare recallers. *Sleep. Med.* **52**, 34–42 (2018). 30218785 10.1016/j.sleep.2018.07.015

[9] Bogdány, T., Perakakis, P., Bódizs, R. & Simor, P. The heartbeat evoked potential is a questionable biomarker in nightmare disorder: a replication study. *NeuroImage: Clin.* **33**, 102933 (2022). 34990964 10.1016/j.nicl.2021.102933 PMC8743245

[10] Otaiku, A. I. Distressing dreams in childhood and risk of cognitive impairment or Parkinson’s disease in adulthood: a national birth cohort study. *eClinicalMedicine* **57**, 101872 (2023). 37064510 10.1016/j.eclinm.2023.101872 PMC10102896

[11] Otaiku, A. I. Distressing dreams, cognitive decline, and risk of dementia: a prospective study of three population-based cohorts. *eClinicalMedicine* **52**, 101640 (2022). 36313147 10.1016/j.eclinm.2022.101640 PMC9596309

[12] Diekelmann, S. & Born, J. The memory function of sleep. *Nat. Rev. Neurosci.* **11**, 114–126 (2010). 20046194 10.1038/nrn2762

[13] Tononi, G. & Cirelli, C. Sleep function and synaptic homeostasis. *Sleep. Med. Rev.* **10**, 49–62 (2006). 16376591 10.1016/j.smrv.2005.05.002

[14] Brodt, S., Inostroza, M., Niethard, N. & Born, J. Sleep—a brain-state serving systems memory consolidation. *Neuron* **111**, 1050–1075 (2023). 37023710 10.1016/j.neuron.2023.03.005

[15] Helfrich, R. F., Mander, B. A., Jagust, W. J., Knight, R. T. & Walker, M. P. Old brains come uncoupled in sleep: slow wave-spindle synchrony, brain atrophy, and forgetting. *Neuron* **97**, 221–230.e224 (2018). 29249289 10.1016/j.neuron.2017.11.020 PMC5754239

[16] Wüst, L. N. et al. Interrelations and functional roles of key oscillatory activities during daytime sleep in older adults. *J. Sleep. Res.* **33**, e13981 (2024). 37488062 10.1111/jsr.13981

[17] Ladenbauer, J. et al. Promoting sleep oscillations and their functional coupling by transcranial stimulation enhances memory consolidation in mild cognitive impairment. *J. Neurosci.* **37**, 7111–7124 (2017). 28637840 10.1523/JNEUROSCI.0260-17.2017 PMC6705731

[18] Kim, J., Gulati, T. & Ganguly, K. Competing roles of slow oscillations and delta waves in memory consolidation versus forgetting. *Cell* **179**, 514–526.e513 (2019). 31585085 10.1016/j.cell.2019.08.040 PMC6779327

[19] Langille, J. J. Remembering to forget: a dual role for sleep oscillations in memory consolidation and forgetting. *Front. Cell. Neurosci.* **13**, 71 (2019). 30930746 10.3389/fncel.2019.00071 PMC6425990

[20] Terzano, M. G. et al. Atlas, rules, and recording techniques for the scoring of cyclic alternating pattern (CAP) in human sleep. *Sleep. Med.* **3**, 187–199 (2002). 14592244 10.1016/s1389-9457(02)00003-5

[21] Colrain, I. M. The K-complex: a 7-decade history. *Sleep* **28**, 255–273 (2005). 16171251 10.1093/sleep/28.2.255

[22] Cox, R., Schapiro, A. C., Manoach, D. S. & Stickgold, R. Individual differences in frequency and topography of slow and fast sleep spindles. *Front. Hum. Neurosci.* **11**, 433 (2017). 28928647 10.3389/fnhum.2017.00433 PMC5591792

[23] Parrino, L. et al. Atlas and updated rules for the scoring of cyclic alternating pattern (CAP) in human sleep. A consensus report by a taskforce of the European Sleep Research Society. *J. Sleep Res*. e70283, 10.1111/jsr.70283 (2026). 10.1111/jsr.70283 PMC13357963 41560629

[24] Parrino, L., Ferri, R., Bruni, O. & Terzano, M. G. Cyclic alternating pattern (CAP): The marker of sleep instability. *Sleep. Med. Rev.* **16**, 27–45 (2012). 21616693 10.1016/j.smrv.2011.02.003

[25] Halász, P. K-complex, a reactive EEG graphoelement of NREM sleep: an old chap in a new garment. *Sleep. Med. Rev.* **9**, 391–412 (2005). 16122950 10.1016/j.smrv.2005.04.003

[26] Terzano, M. G. & Parrino, L. Origin and significance of the cyclic alternating pattern (CAP). *Sleep. Med. Rev.* **4**, 101–123 (2000). 12531162 10.1053/smrv.1999.0083

[27] Biabani, N. et al. Disordered descent into sleep: microstructural divergence across arousal-linked conditions. *npj Biol. Timing Sleep.* **3**, 8 (2026).

[28] Biabani, N. et al. Mapping nocturnal arousal across sleep and pain disorders. *Sci. Rep.* **16**, 8668 (2026). 41786927 10.1038/s41598-026-42639-0 PMC12979684

[29] Marquis, L. P., Paquette, T., Blanchette-Carrière, C., Dumel, G. & Nielsen, T. REM sleep theta changes in frequent nightmare recallers. *Sleep* **40**, zsx110 (2017). 28651358 10.1093/sleep/zsx110 PMC5806577

[30] Sateia, M. J. International classification of sleep disorders-third edition. *Chest* **146**, 1387–1394 (2014). 25367475 10.1378/chest.14-0970

[31] O’Reilly, C., Gosselin, N., Carrier, J. & Nielsen, T. Montreal Archive of Sleep Studies: an open-access resource for instrument benchmarking and exploratory research. *J. Sleep. Res.* **23**, 628–635 (2014). 24909981 10.1111/jsr.12169

[32] World Medical Association World Medical Association Declaration of Helsinki: ethical principles for medical research involving human participants. *JAMA* **333**, 71–74 (2025). 39425955 10.1001/jama.2024.21972

[33] Berry, R. B. et al. Rules for scoring respiratory events in sleep: update of the 2007 AASM manual for the scoring of sleep and associated events. *J. Clin. Sleep. Med.* **8**, 597–619 (2012). 23066376 10.5664/jcsm.2172 PMC3459210

[34] Silber, M. H. et al. The visual scoring of sleep in adults. *J. Clin. Sleep. Med.* **3**, 121–131 (2007). 17557422

[35] Gramfort, A. et al. MEG and EEG data analysis with MNE-Python. *Front. Neurosci.* **7**, 267 (2013). 24431986 10.3389/fnins.2013.00267 PMC3872725

[36] Vallat, R. & Walker, M. P. An open-source, high-performance tool for automated sleep staging. *eLife* **10**, e70092 (2021). 34648426 10.7554/eLife.70092 PMC8516415

[37] Forget, D., Morin, C. M. & Bastien, C. H. The role of the spontaneous and evoked k-complex in good-sleeper controls and in individuals with insomnia. *Sleep* **34**, 1251–1260 (2011). 21886363 10.5665/SLEEP.1250 PMC3157667

[38] Lechat, B., Hansen, K., Catcheside, P. & Zajamsek, B. Beyond K-complex binary scoring during sleep: probabilistic classification using deep learning. *Sleep* **43**, 10.1093/sleep/zsaa077 (2020). 10.1093/sleep/zsaa077 PMC7751135 32301485

[39] Aru, J. et al. Untangling cross-frequency coupling in neuroscience. *Curr. Opin. Neurobiol.* **31**, 51–61 (2015). 25212583 10.1016/j.conb.2014.08.002

[40] Berens, P. CircStat: A MATLAB toolbox for circular statistics. *J. Stat. Softw.* **31**, 1–21 (2009).

[41] Vinck, M., van Wingerden, M., Womelsdorf, T., Fries, P. & Pennartz, C. M. A. The pairwise phase consistency: A bias-free measure of rhythmic neuronal synchronization. *NeuroImage* **51**, 112–122 (2010). 20114076 10.1016/j.neuroimage.2010.01.073

[42] Iacobucci, D., Posavac, S. S., Kardes, F. R., Schneider, M. J. & Popovich, D. L. The median split: Robust, refined, and revived. *J. Consum. Psychol.* **25**, 690–704 (2015).

[43] Ujma, P. P. et al. A comparison of two sleep spindle detection methods based on all night averages: individually adjusted vs. fixed frequencies. *Front. Hum. Neurosci.* **9**, 52 (2015). 25741264 10.3389/fnhum.2015.00052 PMC4330897

