# Supplementary material for: Nightmare disorder shows reduced slow oscillation-dominant spindle coupling in NREM sleep
Source: NPJ Biol Timing Sleep. 2026 Jun 22;3:28. doi: 10.1038/s44323-026-00094-0 (PMC13287732; doi:10.1038/s44323-026-00094-0)
Supplement: Supplementary file 1 — Supplementary Information [file 44323_2026_94_MOESM1_ESM.pdf]

Supplementary Information

Nightmare disorder shows reduced slow oscillation-dominant spindle coupling in NREM sleep

Supplementary Tables

Supplementary Table S1. Oscillatory metrics and definitions. This table defines the key oscillatory metrics used in the manuscript after revision. K-complex measures are normalised to the amount of available N2/N3 sleep where appropriate, to account for stage opportunity.

Metric	Definition	Unit	Used in
V_SO	Mean resultant vector length (MRVL) of slow-oscillation phase at the time of maximal sigma amplitude within each SO event (SO phase band: 0.2-1.25 Hz; sigma amplitude band: 11-16 Hz).	dimensionless; 0-1	Fig. 1; Supplementary Figs S2, S4; Supplementary Tables S4, S8, S11, S15
V_delta	Mean resultant vector length (MRVL) of delta phase at the time of maximal sigma amplitude within each delta event (delta phase band: 0.75-4.25 Hz; sigma amplitude band: 11-16 Hz).	dimensionless; 0-1	Fig. 1; Supplementary Fig. S2; Supplementary Tables S4, S11
CD	Coupling dominance: CD = V_SO - V_delta. Higher values indicate stronger SO-dominant relative to delta-linked spindle coupling.	dimensionless; -1 to +1	Fig. 1; Fig. 3; Supplementary Tables S4, S8, S11, S13-S15
NestRatio	Spindle nesting ratio: NestRatio = N_SO/N_delta, with SO/delta assignment defined by whether a detected spindle centre occurred within +/-0.5 s of an SO or delta peak.	ratio	Results; Supplementary Tables S2-S3; Supplementary Fig. S4
Mean KC density	Subject-level mean K-complex density over 0-6 h from sleep onset, computed as total K-complex count across F3/F4/C3/C4/O1/O2 divided by total available N2/N3 minutes within the same 0-6 h window.	events per available N2/N3 min	Fig. 2b; Supplementary Fig. S1; Supplementary Tables S4, S8, S15
KC_density(t)	Six-channel mean K-complex density trajectory in consecutive 5-min bins from sleep onset, computed from KC count per available N2/N3 minute within each bin and channel. Bins without available N2/N3 sleep were treated as missing for density estimation.	events per available N2/N3 min	Fig. 2a,c
KC_tpeak	Time from sleep onset of the maximum six-channel mean KC_density(t) within the first 0-6 h. This timing metric is reported as a secondary descriptive/sensitivity measure.	hours	Supplementary Figs S6-S7 and S11; Supplementary Tables S4, S8, S15
KC_time_slope	Secondary OLS slope of KC_density(t) versus time from sleep onset over 0-6 h, using bins with finite density.	events min^-1 h^-1	Supplementary Fig. S11; Supplementary Tables S4, S15
CD_age_z	Age-normalised CD: z-scored residuals from control-only regression CD ~ Age.	z-score	Supplementary Table S5
VSO_age_z	Age-normalised V_SO: z-scored residuals from control-only regression V_SO ~ Age.	z-score	Supplementary Table S5
PPC_SO	Pairwise phase consistency for SO-linked coupling, used as a bias-resistant complement to MRVL.	approximately 0-1	Supplementary Tables S12-S14
PPC_delta	Pairwise phase consistency for delta-linked coupling.	approximately 0-1	Supplementary Table S12
PPC_CD	PPC-based dominance index: PPC_CD = PPC_SO - PPC_delta.	approximately -1 to +1	Supplementary Tables S12-S14
N_TotalSpindles_AllChannels	Total detected spindles per subject across F3/F4/C3/C4/O1/O2 during N2+N3 sleep.	count	Supplementary Tables S7, S11
N_SO	Count of spindles assigned to SO events within the specified nesting window.	count	Supplementary Tables S2, S3, S12
N_delta	Count of spindles assigned to delta events within the specified nesting window.	count	Supplementary Tables S2, S3, S12
Prop_nested_any	Proportion of frontal spindles nested in either SO or delta events: (N_SO + N_delta)/N_TotalSpindles_FrontCh.	0-1	Supplementary Table S12

Prop_unnested	Proportion of frontal spindles not nested in SO or delta events: 1 - Prop_nested_any.	0-1	Supplementary Table S12
LowDelta / HighDelta	Within-subject delta-power strata used for state-dependence analyses; metrics recomputed within each stratum.	context label	Supplementary Table S3; Supplementary Fig. S4

Supplementary Table S2. Frontal SO/delta nesting ratio. Frontal slow-oscillation/delta nesting ratio (NestRatio = N_SO/N_delta), computed from subject-level frontal ratios averaged across F3/F4 channels.

Group	Mean ± SD	p-value (Welch t)	Cohen's d (Nightmare vs Control)
Control	0.274 ± 0.105		
Patient	0.247 ± 0.104	0.342	-0.25

Supplementary Table S3. Frontal V_SO and NestRatio in low- versus high-delta contexts. Subject-level frontal SO-spindle coupling strength (V_SO) and SO/delta nesting ratio (NestRatio = N_SO/N_delta) in low- and high-delta epochs.

Group	Metric	Low δ	High δ	High − Low	p-value	Cohen's d
Control	V_SO	0.448 ± 0.136	0.455 ± 0.130	0.006	7.35e-01	0.06
Control	NestRatio	0.188 ± 0.109	0.318 ± 0.119	0.131	1.14e-07	1.21
Patients	V_SO	0.437 ± 0.147	0.393 ± 0.142	-0.045	2.25e-01	-0.24
Patients	NestRatio	0.131 ± 0.062	0.298 ± 0.131	0.167	7.93e-08	1.47

Supplementary Table S4. Group differences in frontal coupling metrics and corrected K-complex summaries. Frontal coupling metrics were averaged across F3/F4. K-complex density is the subject-level mean of six-channel KC counts normalised by available N2/N3 minutes over 0-6 h from sleep onset. KC_tpeak and KC_time_slope are secondary timing metrics.

Metric	Controls	Patients	Cohen's d	p-value
Frontal CD	0.238 ± 0.096	0.137 ± 0.083	-1.121	6.79 × 10^-5
Frontal V_SO	0.443 ± 0.127	0.356 ± 0.136	-0.659	0.0165
Frontal V_delta	0.204 ± 0.079	0.219 ± 0.112	0.160	0.561
Mean KC density (0-6 h; events per available N2/N3 min)	0.723 ± 0.475	0.463 ± 0.345	-0.616	0.019
KC_tpeak (h from sleep onset; secondary)	1.63 ± 1.11	1.97 ± 1.40	0.268	0.326
KC_time_slope (events/min/h; secondary)	-0.143 ± 0.158	-0.095 ± 0.144	0.312	0.238

Supplementary Table S5. Age-normalised SO-dominance in patients. Age-normalised indices for frontal CD and V_SO in nightmare-disorder patients. Values are means +/- SD of z-scores relative to control-only age trends.

Index	Mean ± SD (patients)	Proportion ≤ −1 SD	Proportion ≤ −1.5 SD / ≤ −2 SD
CD_age_z	-0.9 ± 0.9	42.3%	23.1% / 7.7%
VSO_age_z	-0.5 ± 1.2	34.6%	19.2% / 11.5%

Supplementary Table S6. Acquisition and harmonisation details. Cohort-specific acquisition parameters and the common offline harmonisation pipeline used before event detection, coupling analyses, K-complex analyses and PSD estimation.

Parameter	Nightmare disorder cohort (GSTT clinical PSG)	Control cohort (MASS archive)	Harmonisation / analysis handling
Recording environment	Hospital-based clinical sleep laboratory; standard overnight diagnostic PSG.	Laboratory PSG recordings from the Montreal Archive of Sleep Studies (MASS).	Retrospective case-control comparison; site and group are confounded and acknowledged as a limitation.

PSG system / amplifier	Standard clinical PSG systems used at the GSTT Sleep Disorders Centre; exact amplifier models varied across acquisition years.	Standard PSG systems used in contributing MASS recordings; exact amplifier models may vary by source protocol.	All raw EDFs were imported into a common analysis pipeline before event and spectral analyses.
Original EEG montage	Clinical 10-20 PSG montage including F3/F4/C3/C4/O1/O2, bilateral EOG, chin EMG, ECG and respiratory channels.	Comparable PSG montage with the homologous EEG channels required for the present analyses.	Final analyses restricted to shared channels F3, F4, C3, C4, O1 and O2.
Original reference at acquisition	M1/M2 mastoid reference.	Linked-ear reference (LER), as supplied in MASS documentation.	All EEG channels were re-referenced offline to averaged mastoids for harmonised analysis.
Hardware high-pass at acquisition	Typically 0.3 Hz in the analysed clinical recordings.	0.1 Hz.	Common offline zero-phase FIR band-pass of 0.2-35 Hz applied before analysis.
Hardware low-pass at acquisition	Typically 35 Hz in the analysed clinical recordings.	100 Hz.	Common offline zero-phase FIR band-pass of 0.2-35 Hz applied before analysis.
Native EEG sampling rate	Clinical acquisition at >=256 Hz; recordings with higher native rates were resampled.	256 Hz in the MASS recordings used here.	All analysed EEG data were represented at 256 Hz.
Sampling rate used for analysis	256 Hz.	256 Hz.	Common sampling rate used for event detection, coupling and PSD analyses.
Notch filtering	50 Hz.	60 Hz.	Local mains-frequency notch filtering was applied as appropriate.
Downsampling / anti-aliasing	Anti-alias filtering and resampling were applied where native sampling exceeded 256 Hz.	No additional downsampling beyond the common 256 Hz rate.	Implemented within the same Python/MNE-based processing pipeline.
Preprocessing / analysis software	Unified Python/MNE-based pipeline for rereferencing, filtering, downsampling, artefact screening and event/spectral analyses.	Same harmonised Python/MNE-based pipeline.	The same offline settings were used for both cohorts after import.
Sleep scoring / QC	Thirty-second epoch scoring according to AASM criteria by experienced technologists/clinical physiologists with clinical QC.	Source sleep-stage labels from MASS were used after harmonisation; source recordings were manually scored by trained scorers according to the database protocols.	Analyses used harmonised NREM/REM labels; residual scoring/site differences are acknowledged as a limitation.
Artefact handling	Automated artefact screening (flatlines, saturations and extreme amplitude) followed by visual QC.	Same harmonised artefact screening and QC strategy.	Channels/segments failing QC were excluded according to the common pipeline.
Channels used in final analyses	F3, F4, C3, C4, O1, O2.	F3, F4, C3, C4, O1, O2.	Homologous channels only.
K-complex time reference	Sleep onset; 5-min bins across the first 0-6 h.	Sleep onset; 5-min bins across the first 0-6 h.	KC density expressed per minute of available N2/N3 sleep within each bin.
PSD estimator	Welch method; 4-s Hann windows; 50% overlap; constant detrend; density scaling.	Same.	PSD curves reported descriptively for 0.5-30 Hz in NREM and REM.

Supplementary Table S7. Total spindle burden across all EEG channels. Total spindle burden across F3/F4/C3/C4/O1/O2 in N2+N3 sleep.

Group	Mean ± SD	n	Welch t (p-value)	Cohen's d
Control	8037 ± 1453	32		
Patients	10169 ± 2580	26	-3.76 (p = 0.001)	1.05

Supplementary Table S8. Sensitivity of primary frontal coupling metrics and K-complex summaries to prespecified exclusion. Values compare the full sample with a restricted nightmare-disorder sample excluding one participant with substantial periodic limb movements and/or heavy sedative/psychotropic medication use. Values are mean +/- SD; Welch tests are two-sided; Cohen's d is patient minus control.

Metric	Condition	Control mean ± SD	Patients mean ± SD	n (ND)	t (Welch)	p-value	Cohen's d
CD	Full sample	0.238 ± 0.096	0.137 ± 0.083	26	3.96	6.79e-05	-1.121
CD	No PLMS/heavy meds (excl. ND_05)	0.238 ± 0.096	0.137 ± 0.085	25	3.78	0.00041	-1.059
V_SO	Full sample	0.443 ± 0.127	0.356 ± 0.136	26	2.49	0.0165	-0.659
V_SO	No PLMS/heavy meds (excl. ND_05)	0.443 ± 0.127	0.351 ± 0.135	25	2.56	0.0133	-0.689
Mean KC density	Full sample	0.723 ± 0.475	0.463 ± 0.345	26	2.41	0.019	-0.616
Mean KC density	No PLMS/heavy meds (excl. ND_05)	0.723 ± 0.475	0.475 ± 0.347	25	2.28	0.027	-0.586
KC_tpeak	Full sample	1.63 ± 1.11	1.97 ± 1.40	26	-0.99	0.326	0.268

KC_tpeak	No PLMS/heavy meds (excl. ND_05)	1.63 ± 1.11	1.92 ± 1.41	25	-0.85	0.400	0.233
KC_time_slope	Full sample	-0.143 ± 0.158	-0.095 ± 0.144	26	-1.19	0.238	0.312
KC_time_slope	No PLMS/heavy meds (excl. ND_05)	-0.143 ± 0.158	-0.098 ± 0.147	25	-1.12	0.269	0.296

Supplementary Table S9. Slow and fast spindle parameters (fixed frequency approach). Values are subject-wise N2+N3 sleep stage duration-weighted averages by region.

Region	Metric	Control Mean ± SD	Patient Mean ± SD	p-value	Cohen's d
Frontal (F3/F4)	Slow spindle density (events/min, N2+N3)	3.280 ± 0.830	3.427 ± 0.892	0.522	0.171
Frontal (F3/F4)	Fast spindle density (events/min, N2+N3)	1.487 ± 0.678	2.678 ± 1.174	4.73e-05	1.277
Frontal (F3/F4)	Slow spindle amplitude (µV)	106.732 ± 29.908	128.348 ± 60.074	0.103	0.471
Frontal (F3/F4)	Fast spindle amplitude (µV)	106.078 ± 32.231	142.612 ± 110.834	0.115	0.469
Frontal (F3/F4)	Slow spindle duration (s)	1.343 ± 0.198	1.439 ± 0.196	0.0734	0.482
Frontal (F3/F4)	Fast spindle duration (s)	1.313 ± 0.214	1.474 ± 0.230	0.00835	0.730
Frontal (F3/F4)	Slow spindle peak frequency (Hz)	11.667 ± 0.100	11.700 ± 0.094	0.19	0.348
Frontal (F3/F4)	Fast spindle peak frequency (Hz)	13.248 ± 0.203	13.424 ± 0.244	0.00514	0.788
Central (C3/C4)	Slow spindle density (events/min, N2+N3)	1.834 ± 0.749	2.525 ± 1.007	0.00567	0.791
Central (C3/C4)	Fast spindle density (events/min, N2+N3)	2.811 ± 0.757	3.661 ± 1.104	0.00175	0.916
Central (C3/C4)	Slow spindle amplitude (µV)	95.987 ± 23.302	128.025 ± 107.327	0.147	0.434
Central (C3/C4)	Fast spindle amplitude (µV)	91.561 ± 22.487	113.756 ± 38.259	0.0127	0.726
Central (C3/C4)	Slow spindle duration (s)	1.219 ± 0.150	1.362 ± 0.178	0.00207	0.874
Central (C3/C4)	Fast spindle duration (s)	1.225 ± 0.177	1.433 ± 0.227	0.000398	1.034
Central (C3/C4)	Slow spindle peak frequency (Hz)	11.684 ± 0.099	11.671 ± 0.075	0.569	-0.147
Central (C3/C4)	Fast spindle peak frequency (Hz)	13.611 ± 0.322	13.730 ± 0.315	0.164	0.372
Occipital (O1/O2)	Slow spindle density (events/min, N2+N3)	1.205 ± 0.463	2.205 ± 1.008	4.66e-05	1.322
Occipital (O1/O2)	Fast spindle density (events/min, N2+N3)	2.702 ± 0.862	4.041 ± 1.134	9.87e-06	1.349
Occipital (O1/O2)	Slow spindle amplitude (µV)	70.847 ± 20.941	123.654 ± 235.541	0.265	0.334
Occipital (O1/O2)	Fast spindle amplitude (µV)	67.531 ± 20.270	84.587 ± 34.254	0.0309	0.622
Occipital (O1/O2)	Slow spindle duration (s)	0.989 ± 0.125	1.124 ± 0.195	0.00397	0.843
Occipital (O1/O2)	Fast spindle duration (s)	1.060 ± 0.139	1.248 ± 0.210	0.000316	1.080
Occipital (O1/O2)	Slow spindle peak frequency (Hz)	11.726 ± 0.098	11.662 ± 0.068	0.00525	-0.739
Occipital (O1/O2)	Fast spindle peak frequency (Hz)	13.601 ± 0.302	13.832 ± 0.377	0.0145	0.686

Supplementary Table S10. Slow and fast spindle parameters (individual/adaptive frequency approach). Values are N2+N3 sleep stage duration-weighted averages. Spindle subtype is slow versus fast; regions correspond to frontal, central and occipital channels.

Region	Metric	Control	Patient Mean ± SD	p-value	Cohen's d
Frontal (F3/F4)	Slow spindle density (events/min, N2+N3)	3.986 ± 1.073	4.379 ± 1.485	0.265	0.308
Frontal (F3/F4)	Fast spindle density	0.781 ± 0.902	1.726 ± 1.427	0.00546	0.811
Frontal (F3/F4)	Slow spindle amplitude (µV)	106.449 ± 30.066	129.721 ± 64.215	0.0978	0.481
Frontal (F3/F4)	Fast spindle amplitude (µV)	116.789 ± 40.980	235.299 ± 539.374	0.274	0.328
Frontal (F3/F4)	Slow spindle duration (s)	1.351 ± 0.205	1.462 ± 0.203	0.0427	0.547

Frontal (F3/F4)	Fast spindle duration (s)	1.258 ± 0.184	1.454 ± 0.250	0.00175	0.907
Frontal (F3/F4)	Slow spindle peak frequency (Hz)	11.883 ± 0.294	11.966 ± 0.421	0.399	0.233
Frontal (F3/F4)	Fast spindle peak frequency	14.219 ± 0.953	14.173 ± 1.015	0.862	-0.046
Central (C3/C4)	Slow spindle density	3.102 ± 1.588	3.538 ± 1.863	0.348	0.254
Central (C3/C4)	Fast spindle density	1.543 ± 1.450	2.647 ± 1.878	0.0177	0.667
Central (C3/C4)	Slow spindle amplitude (µV)	94.397 ± 23.093	118.069 ± 58.934	0.0626	0.551
Central (C3/C4)	Fast spindle amplitude (µV)	96.472 ± 25.009	142.933 ± 151.443	0.134	0.452
Central (C3/C4)	Slow spindle duration (s)	1.237 ± 0.158	1.383 ± 0.189	0.00271	0.850
Central (C3/C4)	Fast spindle duration (s)	1.139 ± 0.185	1.422 ± 0.303	0.00016	1.156
Central (C3/C4)	Slow spindle peak frequency (Hz)	12.163 ± 0.543	12.052 ± 0.505	0.425	-0.210
Central (C3/C4)	Fast spindle peak frequency	14.385 ± 0.772	14.309 ± 0.881	0.732	-0.092
Occipital (O1/O2)	Slow spindle density	2.363 ± 1.482	3.319 ± 2.054	0.0531	0.543
Occipital (O1/O2)	Fast spindle density	1.544 ± 1.481	2.927 ± 2.068	0.00635	0.783
Occipital (O1/O2)	Slow spindle amplitude (µV)	69.360 ± 20.297	90.710 ± 64.098	0.113	0.470
Occipital (O1/O2)	Fast spindle amplitude (µV)	72.952 ± 21.852	132.086 ± 257.132	0.253	0.343
Occipital (O1/O2)	Slow spindle duration (s)	1.018 ± 0.136	1.144 ± 0.205	0.0103	0.740
Occipital (O1/O2)	Fast spindle duration (s)	0.992 ± 0.140	1.232 ± 0.276	0.00028	1.134
Occipital (O1/O2)	Slow spindle peak frequency (Hz)	12.225 ± 0.557	12.092 ± 0.580	0.381	-0.234
Occipital (O1/O2)	Fast spindle peak frequency (Hz)	14.394 ± 0.783	14.347 ± 0.833	0.827	-0.058

Supplementary Table S11. Robustness of frontal coupling group effects to all-channel spindle burden and extreme spindle counts. Frontal coupling metrics are subject-level means averaged across F3/F4.

Analysis	CD (Control)	CD (Nightmare)	p (CD)	V_SO (Control)	V_SO (Patient)
Primary analysis	0.238 ± 0.096	0.137 ± 0.083	6.79e-05	0.443 ± 0.127	0.356 ± 0.136
Exclude extremes (5% tails of N_TotalSpindles)	0.242 ± 0.094	0.134 ± 0.085	7.89e-05	0.446 ± 0.127	0.341 ± 0.126
Adjusted model (Group + N_TotalSpindles + Age + Sex)	β_group = -0.108		2.59e-04	β_group = -0.098	p = 0.014

Supplementary Table S12. Robustness (PPC) and spindle nesting proportions in frontal channels. Proportions are computed per channel and averaged within subject; pooled frontal counts are additionally reported for interpretability.

Metric	Controls mean ± SD (n)	Patient	p (Welch)	Cohen's d
PPC_SO	0.206 ± 0.116 (n=32)	0.134 ± 0.102 (n=26)	0.0143	-0.66
PPC_Delta	0.046 ± 0.035 (n=32)	0.058 ± 0.054 (n=26)	0.328	0.27
PPC_CD	0.160 ± 0.095 (n=32)	0.075 ± 0.068 (n=26)	0.000223	-1.03
Prop_nested_any	0.394 ± 0.037 (n=32)	0.290 ± 0.059 (n=26)	1.66e-09	-2.10
Prop_unnested	0.606 ± 0.037 (n=32)	0.710 ± 0.059 (n=26)	1.66e-09	2.10
N_unnested (count)	1689 ± 344 (n=32)	2378 ± 601 (n=26)	7.19e-06	1.41

Supplementary Table S13. Age- and sex-matched resampling sensitivity analysis. Controls were repeatedly sampled with replacement to match the nightmare group on sex and age proximity across 10,000 iterations.

Outcome	Controls	Patient	Full-sample Δ (Nightmare - Control)	Cohen's d	Full-sample p (Welch)	Matched Δ (median [95% interval])	Matched d (median [95% interval])	Direction consistent	Resamples p<0.05
Frontal CD (V_SO - V_δ)	0.238 ± 0.096	0.137 ± 0.083	-0.101	-1.12	6.79×10-5	-0.081 [-0.114, -0.050]	-0.90 [-1.28, -0.56]	100%	97.6%
Frontal PPC_CD (PPC_SO - PPC_δ)	0.160 ± 0.095	0.075 ± 0.068	-0.084	-1.01	2.23×10-4	-0.062 [-0.091, -0.036]	-0.76 [-1.07, -0.48]	100%	91.6%

Supplementary Table S14. Event-count matched resampling sensitivity analysis. Controls were repeatedly sampled with replacement to match the nightmare group on sex and coupling-event counts (n_SO and n_delta) across 10,000 iterations.

Outcome	Controls mean ± SD	Patient mean ± SD	Full-sample Δ (Nightmare - Control)	Cohen's d	Full-sample p (Welch)	Matched Δ (median [95% interval])	Matched d (median [95% interval])	Direction consistent	Resamples p<0.05
Frontal CD (V_SO - V_δ)	0.238 ± 0.096	0.137 ± 0.083	-0.101	-1.12	6.79e-05	-0.086 [-0.116, -0.056]	-1.04 [-1.48, -0.66]	100.0%	99.3%
Frontal V_SO	0.443 ± 0.127	0.356 ± 0.136	-0.086	-0.66	1.65e-02	-0.076 [-0.119, -0.031]	-0.59 [-0.95, -0.25]	99.9%	57.6%
Frontal PPC_CD (PPC_SO - PPC_δ)	0.160 ± 0.095	0.075 ± 0.068	-0.084	-1.01	2.23e-04	-0.072 [-0.103, -0.042]	-0.95 [-1.36, -0.58]	100.0%	98.3%

Supplementary Table S15. Multivariable robustness models. Values are patient-minus-control coefficients from linear models. Site could not be included because group and site were perfectly confounded.

Outcome	Model covariates	Group coef (Nightmare vs Control)	SE	p
CD (frontal)	Age + Sex	-0.097	0.026	4.61e-04
CD (frontal)	Age + Sex + N2% + N3% + REM%	-0.098	0.032	0.003
CD (frontal)	Age + Sex + N2N3%	-0.112	0.027	9.81e-05
CD (frontal)	Age + Sex + N2N3% + arousal + sleep efficiency	-0.095	0.033	0.007
CD (frontal)	Age + Sex + N2% + N3% + REM% + arousal + sleep efficiency	-0.088	0.036	0.020
Mean KC density (events per available N2/N3 min)	Age + Sex	-0.387	0.112	0.00107
Mean KC density (events per available N2/N3 min)	Age + Sex + N2N3%	-0.426	0.117	0.00063
KC_tpeak (h; secondary)	Age + Sex	0.521	0.354	0.146
KC_tpeak (h; secondary)	Age + Sex + N2N3%	0.474	0.374	0.21
KC_time_slope (events/min/h; secondary)	Age + Sex	0.079	0.042	0.0686
KC_time_slope (events/min/h; secondary)	Age + Sex + N2N3%	0.096	0.044	0.0346

Supplementary Figures

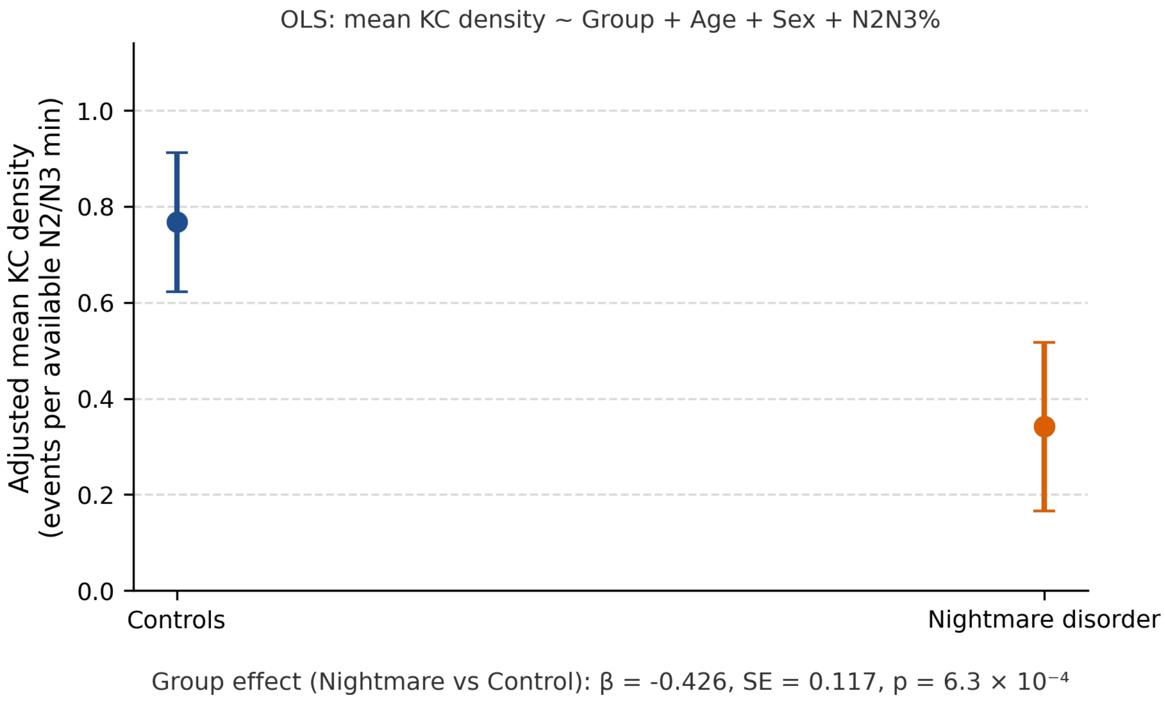

Supplementary Figure S1. Multivariable-adjusted mean K-complex density. Estimated marginal means and 95% confidence intervals are shown from an OLS model of subject-level mean K-complex density over 0-6 h from sleep onset with Group, Age, Sex and N2N3% as predictors. Mean K-complex density was computed as total K-complex count across F3/F4/C3/C4/O1/O2 divided by total available N2/N3 minutes within the same 0-6 h window. The group coefficient was lower in nightmare disorder (beta = -0.426 events per available N2/N3 min, SE = 0.117, p = 6.3 x 10^-4).

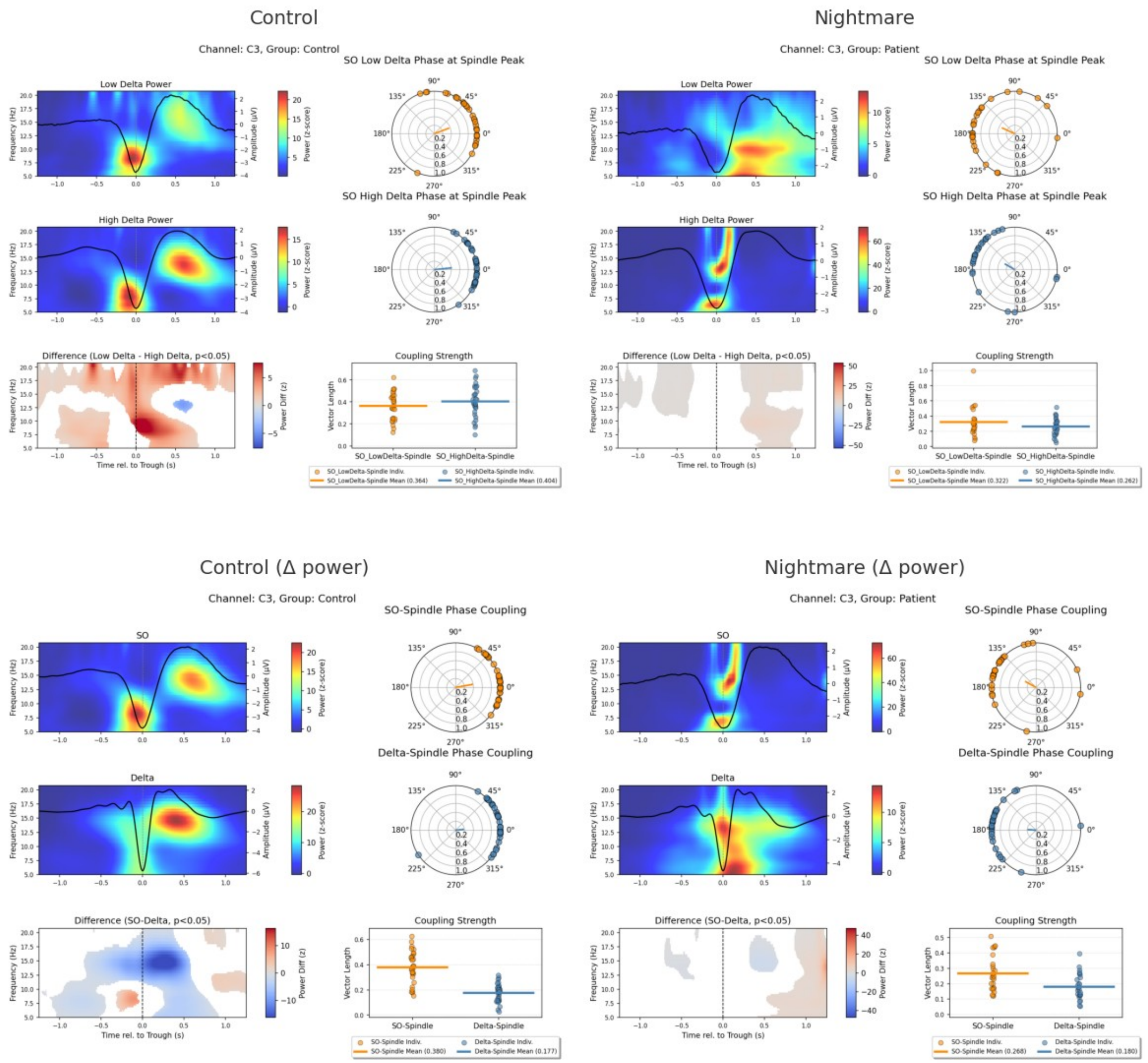

Supplementary Figure S2. Time–frequency and phase–amplitude profiles of slow oscillations, delta waves and spindles at C3 channel. Examples from the central channel C3 illustrating slow-oscillation (SO) and delta-wave dynamics and their coupling to spindle activity in controls (left column) and nightmare-disorder patients (right column). Slow-wave trace shown in standardised units (z) for visualisation. Top row (Low vs High δ context): Event-locked time–frequency representations (TFRs) for SO-locked events during Low δ and High δ background activity, averaged across subjects. Power is expressed as z-scores relative to a pre-event baseline; the overlaid black trace shows the band-pass filtered slow wave. Adjacent polar plots display the distribution of SO phase at spindle-amplitude peak for Low δ and High δ conditions, with resultant vectors indicating mean phase and vector length. Difference maps (Low δ − High δ) and coupling-strength scatter plots (individual V_SO values with group means) are shown below. In controls, C3 SO–spindle coupling is slightly stronger in High δ than Low δ epochs (V_SO 0.44 ± 0.16 vs 0.49 ± 0.18; High − Low = 0.05, p ≈ 0.10), whereas in nightmares the trend is reversed (0.39 ± 0.18 vs 0.34 ± 0.11; High − Low = −0.05, p ≈ 0.15). Bottom row (SO vs delta waves): TFRs for SO-locked and delta-locked events, accompanied by SO–spindle and delta–spindle phase histograms and resultant vectors, difference maps (SO − Delta) and coupling-strength scatter plots. In controls, SO-locked events exhibit a pronounced spindle-band power increase and strong SO–spindle coupling at the up-state (V_SO 0.47 ± 0.16) compared with weaker delta–spindle coupling (V_δ 0.24 ± 0.08), yielding a frontal-centred coupling dominance CD ≈ 0.23 ± 0.14. In nightmare-disorder patients, SO–spindle coupling at C3 is reduced (V_SO 0.33 ± 0.12) while delta–spindle coupling remains similar (V_δ 0.24 ± 0.10), resulting in a markedly lower CD ≈ 0.09 ± 0.11 (t ≈ −4.33, p ≈ 6.2×10^{-5}, Cohen's d ≈ −1.11). These C3 profiles illustrate at a single site the broader group-level pattern reported in Figure 1, namely a relative weakening of SO-dominant spindle coupling in nightmare disorder with preserved delta–spindle engagement.

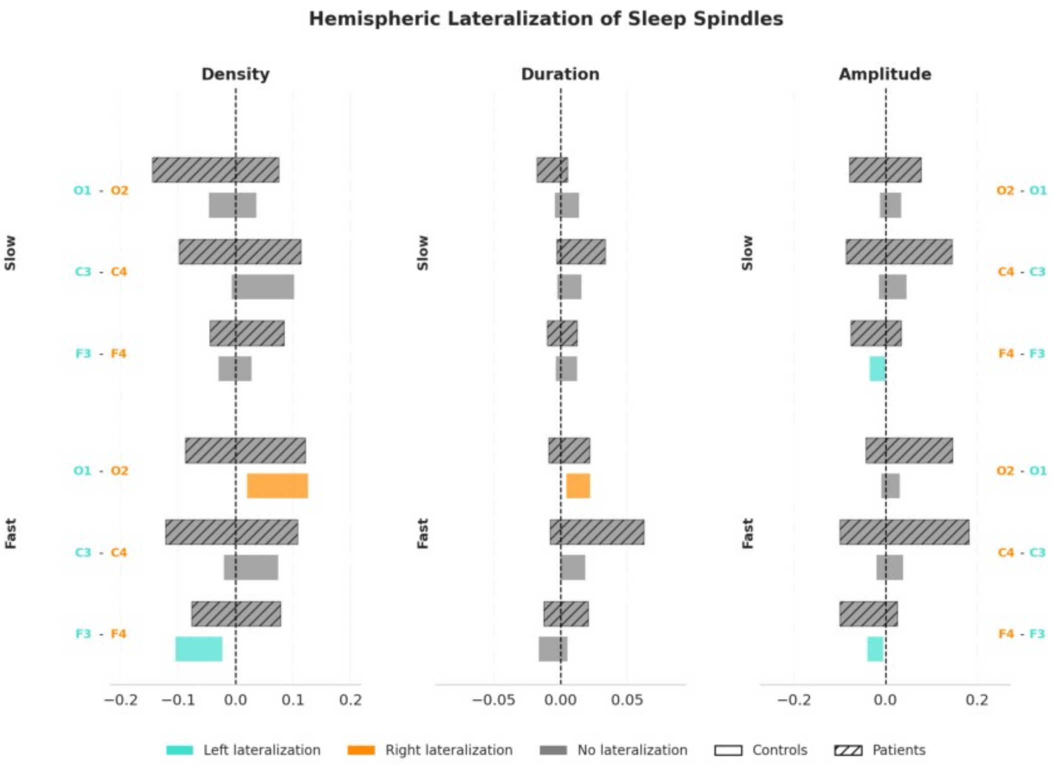

Supplementary Figure S3. Hemispheric lateralisation of sleep spindles. Hemispheric lateralisation indices for slow and fast spindles across frontal (F3–F4), central (C3–C4) and occipital (O1–O2) regions. For each subject and channel pair, a lateralisation index was computed for spindle density (events/min in N2+N3), mean duration and mean peak-to-peak amplitude; bars show group means for controls (solid grey) and nightmare-disorder patients (hatched) with direction coded as left-lateralised (turquoise), right-lateralised (orange) or near-symmetric (grey).

In both groups, slow spindle density exhibits the expected modest left-lateralisation frontally and central symmetry, whereas occipital slow spindles show a small right bias. Fast spindles show a similar pattern with slightly stronger lateralisation in central and occipital pairs. Duration and amplitude indices cluster close to zero in most regions, indicating only weak hemispheric asymmetries. Importantly, controls and nightmare-disorder patients show broadly overlapping lateralisation profiles across all metrics and regions, with no large or systematic group shifts. These findings support the impression that nightmare disorder affects the overall balance of SO–delta–spindle regimes rather than introducing marked hemispheric asymmetries in spindle generation.

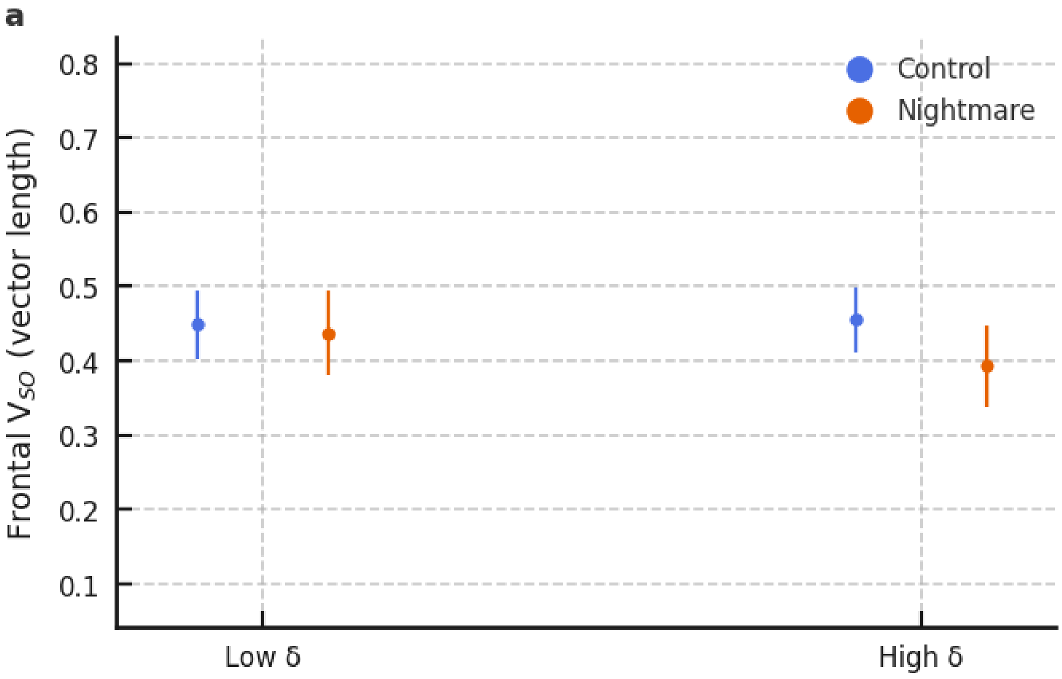

Supplementary Figure S4. Frontal SO–spindle coupling in low- versus high-delta contexts. Frontal slow-oscillation–spindle coupling strength (V_SO, vector length) averaged across F3/F4 and stratified by delta background activity. For each subject, N2/N3 epochs were divided into Low δ and High δ based on within-subject delta power, and V_SO was recomputed separately for SO events occurring in each context. Points (blue, Control; orange, Nightmare) are jittered horizontally around the context positions. Black circles indicate group means with 95% confidence intervals. Across both contexts, nightmare-disorder patients exhibit weaker frontal SO–spindle coupling than controls, consistent with the group differences in overall V_SO and coupling dominance shown in Figure 1. The modest increase in V_SO from Low δ to High δ in both groups suggests that SO–spindle coupling is state-dependent, but the primary abnormality in nightmare disorder is a global reduction in SO coupling rather than a deficit restricted to a particular delta context.

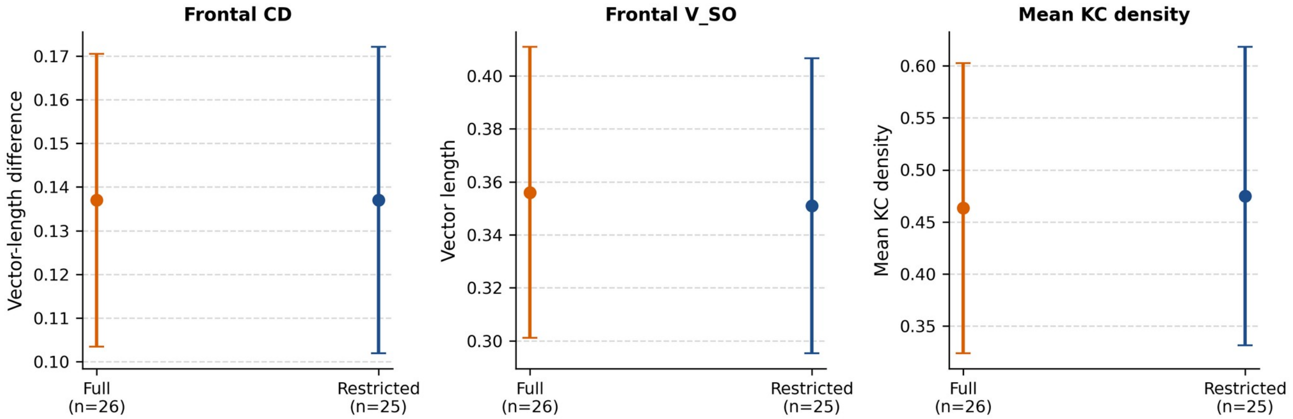

Points show nightmare-disorder means with 95% confidence intervals before and after excluding one participant.

Supplementary Figure S5. Sensitivity of frontal coupling and K-complex density to predefined exclusion. Nightmare-disorder means with 95% confidence intervals are shown for the full sample (n = 26) and after excluding one participant meeting predefined substantial comorbidity/medication criteria (n = 25). Frontal coupling dominance CD and frontal V_SO were subject-level F3/F4 averages. Mean KC density was computed over 0-6 h as total K-complex count across F3/F4/C3/C4/O1/O2 divided by total available N2/N3 minutes in the same window. Exclusion had negligible impact on the main coupling metrics and did not materially change mean K-complex density.

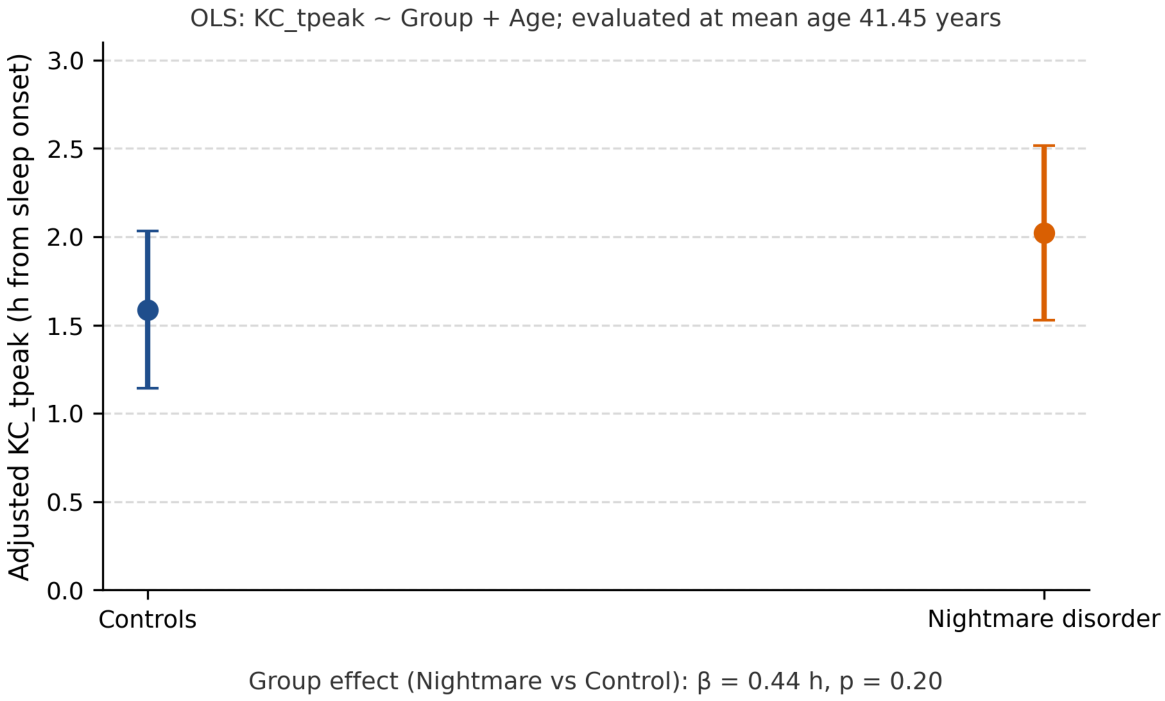

Supplementary Figure S6. Age-adjusted K-complex peak timing. KC_tpeak was defined as the time from sleep onset at which the six-channel mean K-complex density trajectory reached its maximum within the first 0-6 h. Points show age-adjusted group means with 95% confidence intervals from an OLS model, KC_tpeak ~ Group + Age, evaluated at the sample mean age (41.45 years). KC_tpeak was numerically later in nightmare disorder but the age-adjusted group effect was not statistically robust (beta = 0.44 h, p = 0.20).

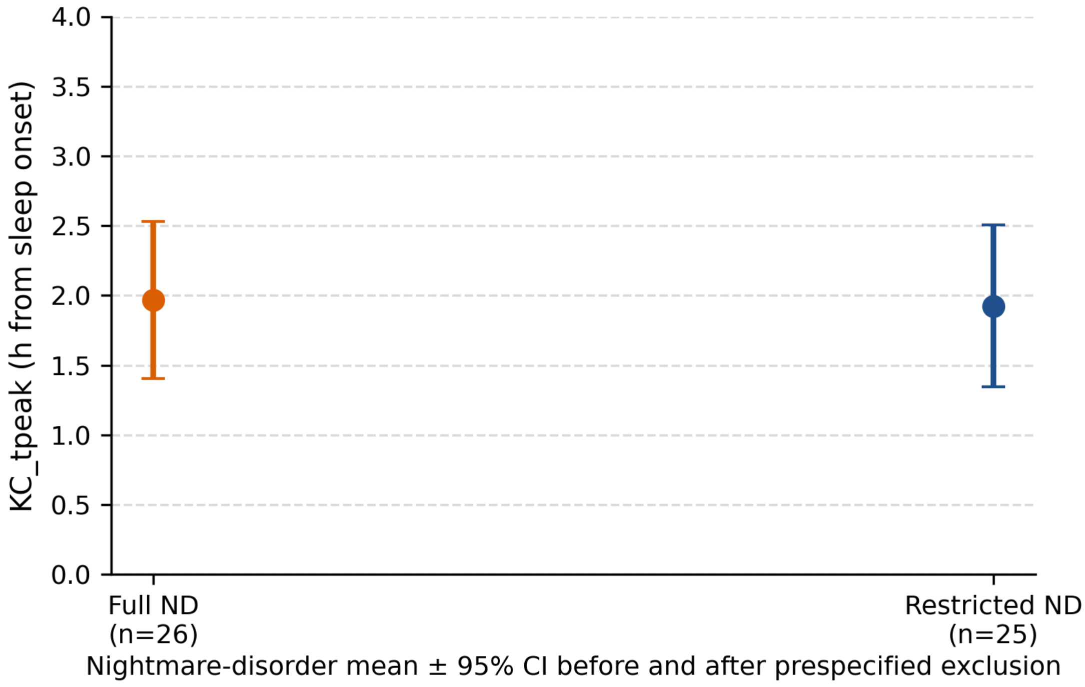

Supplementary Figure S7. Sensitivity of KC_tpeak to prespecified exclusion. Nightmare-disorder KC_tpeak means with 95% confidence intervals are shown in the full sample (n = 26) and after excluding the participant meeting prespecified substantial comorbidity/medication criteria (n = 25). Exclusion did not materially change KC_tpeak (1.97 +/- 1.40 h in the full nightmare sample versus 1.92 +/- 1.41 h after exclusion).

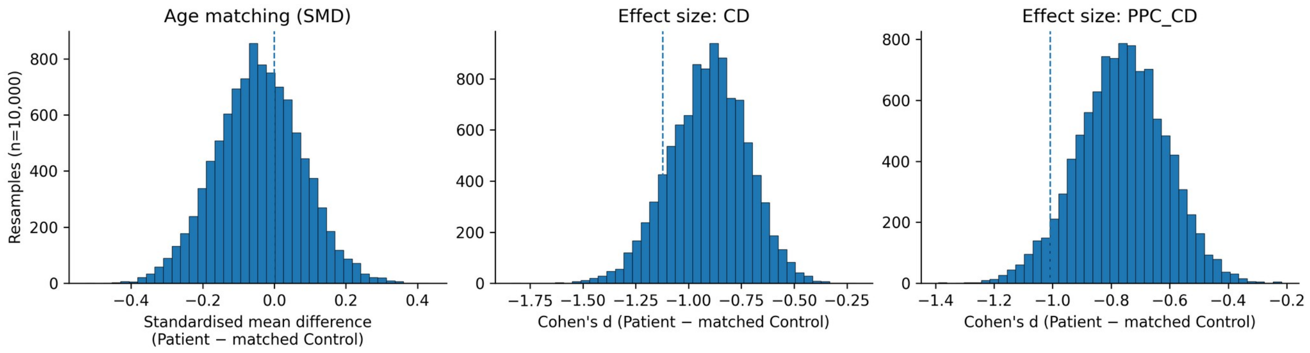

Supplementary Figure S8. Age- and sex-matched resampling sensitivity analysis. To assess whether demographic imbalance could account for the observed group differences, we generated 10,000 age/sex-matched resamples of the control cohort. For each resample, we constructed a matched control sample by sampling with replacement one control per patient, matching sex exactly and preferentially matching age (Gaussian kernel weighting; σ = 10 years). Histograms show the distribution across resamples of (left) age standardised mean difference (patients − matched controls), (middle) Cohen's d for frontal coupling dominance CD, and (right) Cohen's d for PPC-based dominance PPC_CD. Vertical dashed lines indicate the full-sample effect sizes. Across resamples, CD and PPC_CD remained lower in nightmare-disorder patients (see Supplementary Table S13).

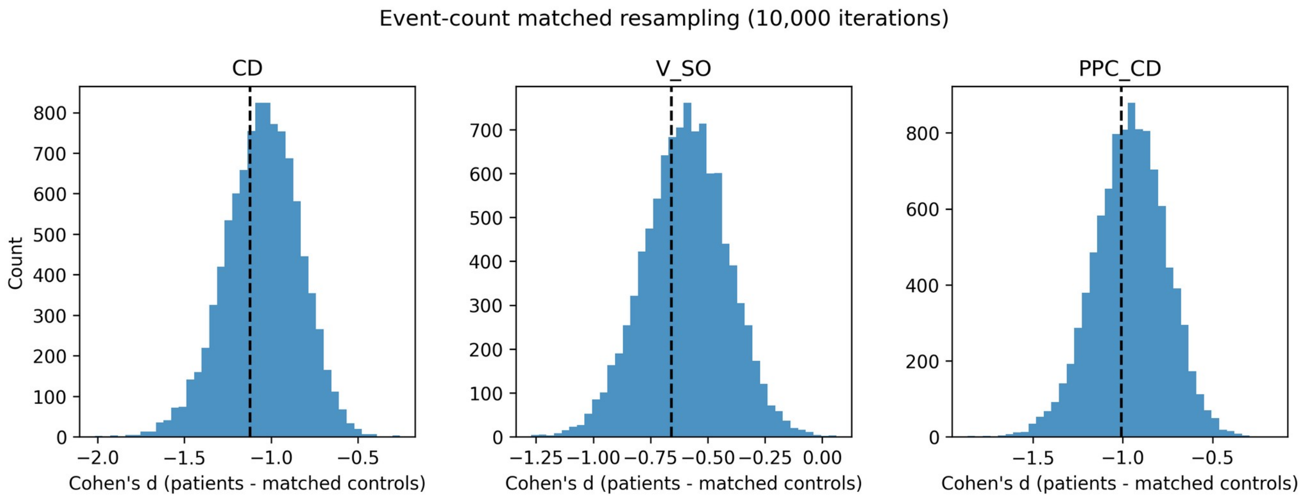

Supplementary Figure S9. Event-count matched resampling sensitivity analysis. Histograms show the distribution of Cohen's d (patient − matched control) across 10,000 resamples, where controls were sampled with replacement to match patients on sex and coupling-event counts (n_SO and n_δ; Gaussian kernel weighting). Dashed vertical lines denote the full-sample Cohen's d.

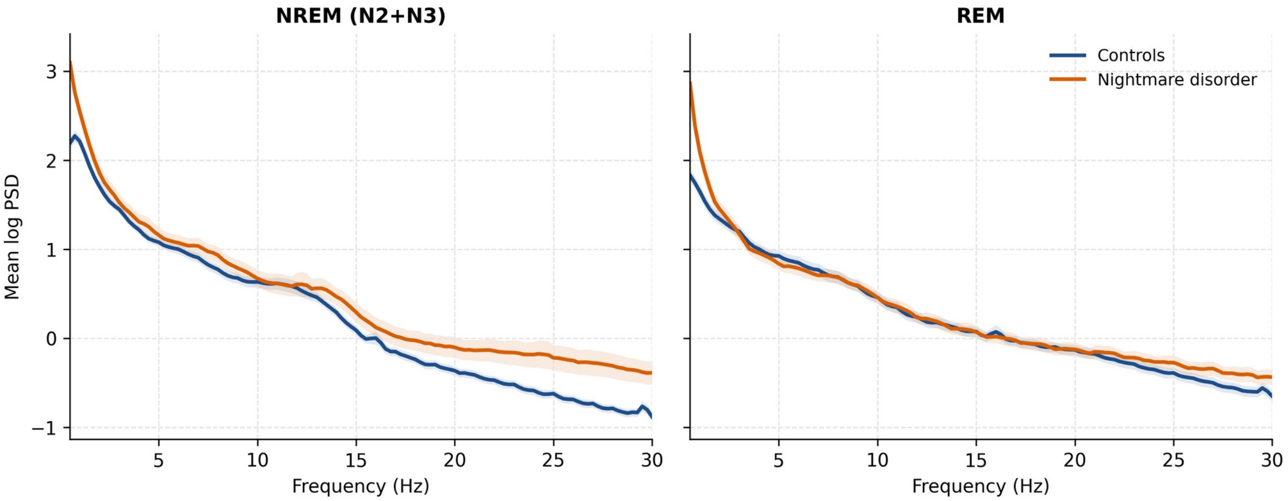

Curves show channel-averaged group mean log PSD across F3/F4/C3/C4/O1/O2; shaded bands show average channel-level SEM.

Supplementary Figure S10. Stage-wise Welch power spectral density overview. Descriptive NREM (N2+N3) and REM spectra are shown for controls and nightmare-disorder patients. Curves represent group mean log-transformed PSD averaged across F3/F4/C3/C4/O1/O2; shaded bands show the average channel-level SEM. PSDs were estimated using the harmonised preprocessing pipeline and Welch method. These spectra are provided as reviewer-requested EEG context and were not treated as primary inferential endpoints because absolute spectral amplitudes are more sensitive to site/acquisition differences than within-recording coupling metrics.

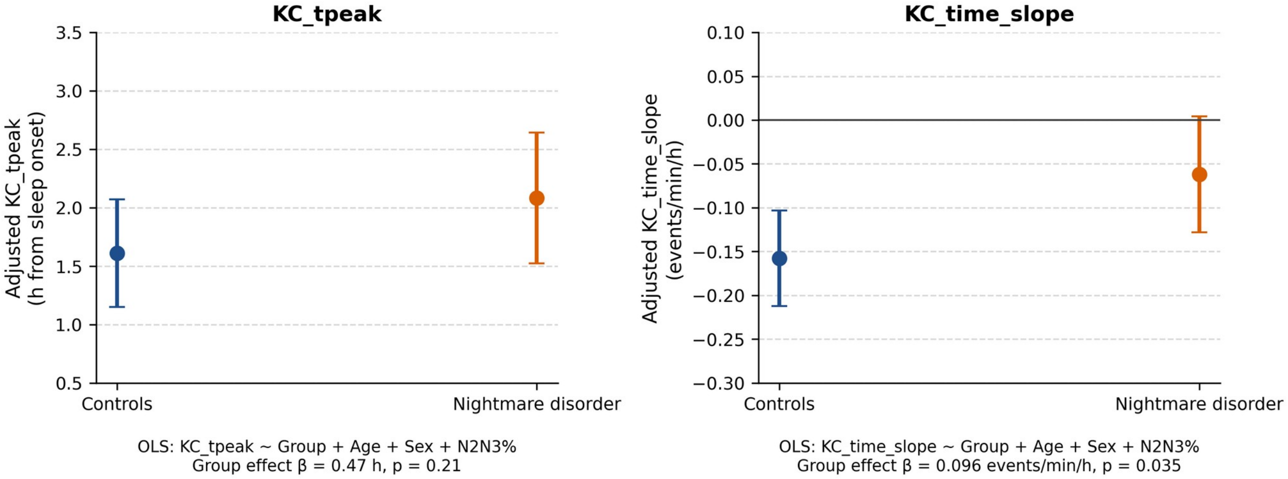

Points indicate estimated marginal means with 95% confidence intervals; models adjust for age, sex and N2N3%.

Supplementary Figure S11. Stage-adjusted K-complex timing metrics. Estimated marginal means and 95% confidence intervals are shown from OLS models adjusting for Age, Sex and N2N3%. KC_tpeak is the time from sleep onset of the maximum six-channel mean KC density within 0-6 h. KC_time_slope is the secondary OLS slope of KC_density(t) versus time from sleep onset over 0-6 h. KC_tpeak was not robustly different between groups (beta = 0.47 h, p = 0.21). KC_time_slope is reported as a secondary timing sensitivity metric (beta = 0.096 events/min/h, p = 0.035) and is not used as the primary K-complex conclusion.
